# Synthesis of Spironucleosides: Past and Future Perspectives

**DOI:** 10.3390/molecules22112028

**Published:** 2017-11-22

**Authors:** Raquel G. Soengas, Gustavo da Silva, Juan Carlos Estévez

**Affiliations:** 1 Organic Chemistry Area, University of Almeria, Carretera de Sacramento s/n, 04120 Almería, Spain; 2 Research Institute for Medicines (iMed.ULisboa), Faculty of Pharmacy, Universidade de Lisboa, 1649-003 Lisbon, Portugal; galfdsilva@ff.ulisboa.pt; 3 Research Center in Biological Chemistry and Molecular Materials (CIQUS) and Organic Chemistry Department, University of Santiago de Compostela, 15782 Santiago de Compostela, Spain

**Keywords:** spironucleosides, spirohydantoins, spirodiketopiperazines, sugar amino acids

## Abstract

Spironucleosides are a type of conformationally restricted nucleoside analogs in which the anomeric carbon belongs simultaneously to the sugar moiety and to the base unit. This locks the nucleic base in a specific orientation around the *N*-glycosidic bond, imposing restrictions on the flexibility of the sugar moiety. Anomeric spiro-functionalized nucleosides have gained considerable importance with the discovery of hydantocidin, a natural spironucleoside isolated from fermentation broths of *Streptomyces hygroscopicus* which exhibits potent herbicidal activity. The biological activity of hydantocidin has prompted considerable synthetic interest in this nucleoside and also in a variety of analogues, since important pharmaceutical leads can be found among modified nucleoside analogues. We present here an overview of the most important advances in the synthesis of spironucleosides.

## 1. Introduction

The study of the chemistry and biochemistry of nucleosides has been a fundamental research field since the crucial role of nucleic acids in cells was established in the 1950s. It was then when the role of nucleic acids as constituents of the macromolecules that convey genetic information in living cells was established. As the metabolic processes involving nucleic acids became understood, the interest in analogues nucleosides grew. In this regard, nucleoside analogues has been a subject of great interest in the development of novel drugs owing to the fact that they can be involved in the disruption of nucleic acid biosynthesis and thus inhibit a series of crucial biological processes, such as cellular division and viral replication [1]. Using this concept, several nucleoside analogues designed to interact with DNA (RNA) and to inhibit enzymes utilizing them possess antiviral, antimetabolic, and antibacterial properties and are currently in use in clinical fields [2,3,4,5,6,7,8,9,10,11].

The extensive search for clinically useful nucleoside derivatives has resulted in a plethora of bioactive modified nucleosides. Taking into account that in many occasions there is a close correlation between reduced conformational flexibility and a potent interaction with biomacromolecules, the modifications include the preparation of conformationally restricted nucleosides. These nucleoside analogues can show a dramatic improvement in enzymatic recognition, as well as enhancing base stacking and backbone pre-organization [12]. Conformational restriction of the furanose ring of nucleosides, nucleotides and oligonucleotides has been intensively pursued in recent years, stimulated by the potential application of these molecules as therapeutic agents [13,14,15,16]. Among those, spiro-functionalized nucleosides have recently gained more interest.

Spironucleosides are a family of conformationally restricted nucleosides in which the anomeric carbon belongs simultaneously to a pyranoid or furanoid sugar ring and to an aza-heterocyclic moiety [17]. This fixes the nucleotide base in a specific orientation around the *N*-glycosidic bond, thus altering the flexibility of the sugar moiety. Anomeric spirocyclic nucleosides gained considerable interest with the discovery of (+)-hydantocidin, a natural spironucleoside isolated from fermentation broths of *Streptomyces hygroscopicus* SANK 63584 [18,19], Tu-2474 [20] and A1491 [21]. Hydantocidin exhibits potent herbicidal and plant growth regulatory activity with high selective toxicity between plants and animals [22] Biochemical studies have shown that hydantocidin is a proherbicide which is phosphorylated at the 5′ position in vivo and inhibits adenylosuccinate synthetase (AdSS) [23], an enzyme that plays an important role in the de novo purine synthesis in plants [24]. These observations have understandably stimulated considerable interest, not only in the synthesis of (+)-hydantocidin itself, but also in a variety of its analogues, with the notion that important pharmaceutical leads can be found among modified nucleoside analogues. A number of anomeric spirocyclic nucleosides have subsequently appeared in the literature being hydantoines or diketopiperazines analogues, but also, barbiturates and more diverse spiroheterocyclic subunits.

This extensive research on the synthesis and biology of hydantocidin analogues was awarded with the discovery of a glucopyranose spirohydantoin which is the most active inhibitor of glycogen phosphorylase (GP) known to date, with a *K_i_* value of 3.1 µM [25]. Glycogen phosphorylase is a key enzyme in the regulation of muscle and hepatic glycogen metabolism, and catalyzes the first step in the intracellular degradation of glycogen [26,27,28,29]. Inhibition of glycogen phosphorylase is believed to assist in shifting the equilibrium between glycogen degradation and glycogen synthesis in favor of the latter in both muscle and liver [30,31,32,33]. Therefore, GP inhibitors may be clinically useful for the treatment of diabetes mellitus, especially the non-insulin dependent diabetes mellitus (NIDDM or type II diabetes) [34,35].

Most of the synthetic strategies for spironucleosides revolve around the use of carbohydrate derivatives to generate the desired stereochemistry in the sugar ring. However, a very diverse range of strategies are available for the synthesis of the characteristic spirocyclic base. Unfortunately, to the best of our knowledge, the literature suffers from the lack of an exhaustive review on the preparation of spironucleosides. Our main aim in this review is to draw together all of the synthetic information on spironucleosides in a form which is easily consulted The coverage is primarily from the point of view of organic chemists, so our intention is to describe in detail those strategies that have been employed to synthetize spiroonucleosides.

## 2. Synthesis of Hydantoins

### 2.1. (+)-Hydantocidin

**Figure 1:**
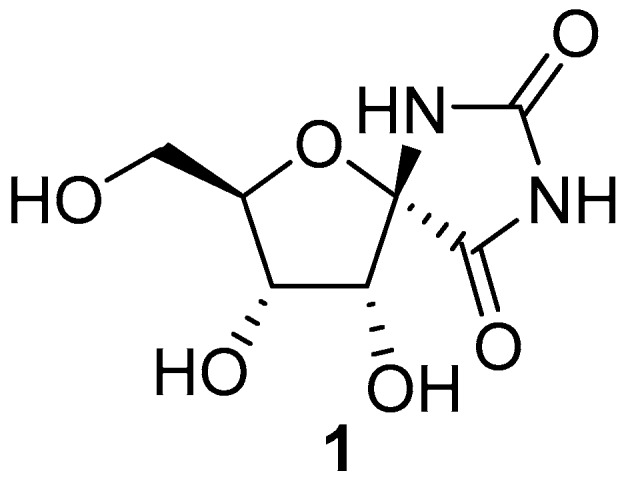
Natural spironucleoside (+)-hydantocidin (**1**).

The most representative member of the hydantoins family is (+)-hydantocidin (**1**, Figure 1), a natural spironucleoside displaying potent herbicidal activity with high selective toxicity between plants and animals.

**Scheme 1:**
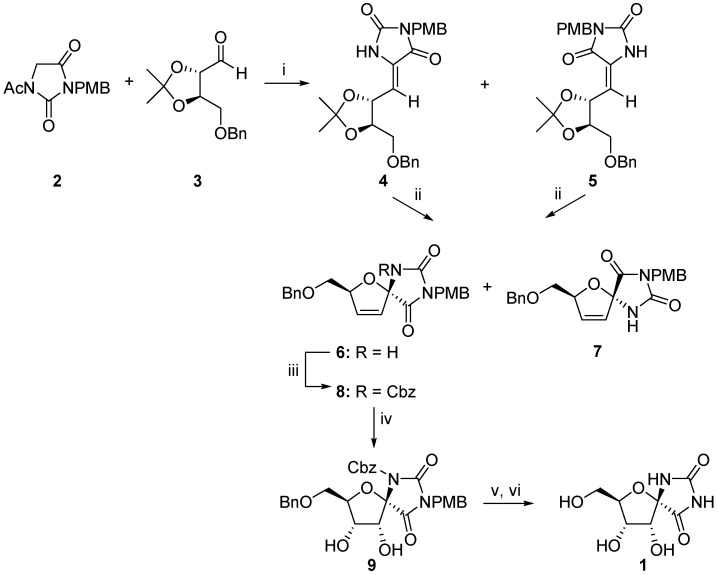
Mio et al. total synthesis of (+)-hydantocidin (**1**). Reagents and conditions: (i) *t*-BuOK, dioxane, r.t., 4 h, 71% of **4** and 14% of **5**; (ii) *p*-TsOH-H_2_O, MS 4Å, reflux, 2 h, 82%, **6**/**7** 63:37; (iii) CbzCl, *t*-BuOK, THF, r.t.; (iv) OsO_4_, *N*-methylmorpholine-*N*-oxide, acetone/H_2_O, r.t., 24 h, 48%; (v) CAN, CH_3_CN/H_2_O, r.t., 20 min, 94%; (vi) H_2_/Pd-C (5%), CH_3_OH, 55 °C, 6 h.

This interesting biological profile prompted many research groups to investigate the synthesis of hydantocidin (**1**). The first total synthesis of **1**, proposed by Mio and co-workers in 1991, included as key step the condensation of a tetrose and a hydantoin ring [36]. Starting from 4-*O*-benzyl-2,3-isopropylidene-d-threose (**3**), aldol condensation with l-*N*-acetyl-3-*N*-(4-methoxybenzyl)hydantoin (**2**) in the presence of potassium *tert*-butoxide afforded a mixture of (*Z*)-isomer **4** and (*E*)-isomer **5** (Scheme 1). Treatment of both isomers **4** and **5** under transketalization conditions led to the mixture of cyclized products **6** and **7**, which were separated by column chromatography. Introduction of a benzyloxycarbonyl group at the amide-NH group of **6**, followed by diastereoselective dihydroxylation of the olefin on the β-face and removal of the protecting groups, finally gave desired (+)-hydantocidin (**1**).

**Scheme 2:**
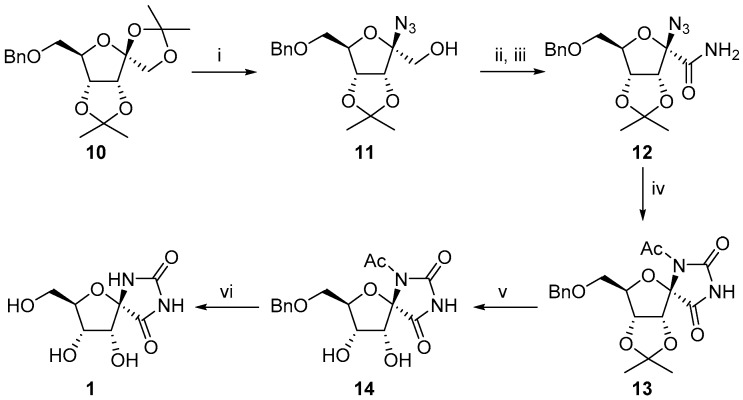
Improved Mio et al. synthesis of (+)-hydantocidin (**1**). Reagents and conditions: (i) TMSN_3_, TMSOTf, CH_3_CN, r.t.; (ii) (COCl)_2_, DMSO, Et_3_N, CH_2_Cl_2_. r.t., 96%; (iii) 1. NaClO_2_, NaH_2_PO_4_, 2-methylbutene, *t*-BuOH/H_2_O, r.t.; 2. ClCO_2_Et, Et_3_N, CH_2_Cl_2_, 0 °C then NH_3_, 72%; (iv) PBu_3_, CO_2_ gas, CH_3_CN, r.t., 5 h then Ac_2_O, DMAP, r.t., 90%; (v) DOWEX 50 (H^+^), MeOH/H_2_O, r.t., 92%; (vi) 1. NH_2_NH_2_, MeOH, r.t.; 2. H_2_, Pd/C, MeOH, 55 °C, 30 min, 88%.

Because of the need for laborious chromatography, this methodology is inconvenient for the large-scale synthesis of (+)-hydantocidin. Mio and co-workers subsequently developed a new synthetic method overcoming these problems [37]. The procedure (Scheme 2) involves the *N*-glycosidation of a d-psicofuranose derivative prior to the formation of the hydantoin ring. Thus, treatment of psicofuranose derivative **10** [38] with azidotrimethylsilane in the presence of catalytic amounts of trimethylsilyltriflate, afforded the desired azide **11**. Oxidation of the primary hydroxyl group to the corresponding carboxylic acid, followed by coupling with ammonia, afforded amide **12**. Reaction of **12** with tributylphosphine in acetonitrile yielded the corresponding spiro-hydantoin, which was immediately acetylated in order to avoid epimerization at the spiro-center to afford **13**. Removal of the protecting groups finally gave desired hydantocidin (**1**).

**Scheme 3:**
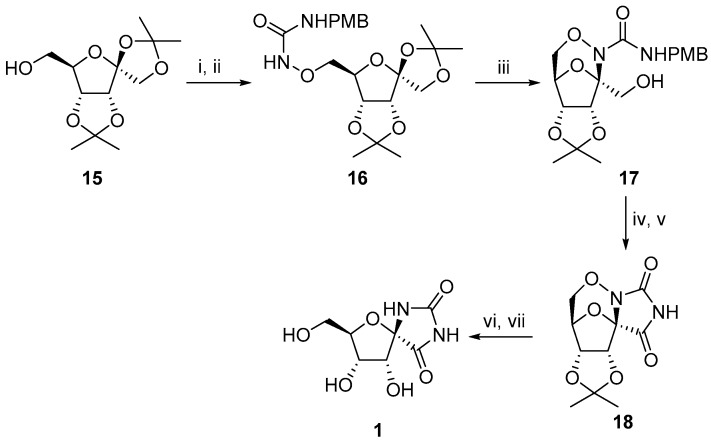
Chemla et al. synthesis of (+)-hydantocidin (**1**). Reagents and conditions: (i) 1. *N*-hydroxy-phtalimide, PPh_3_, DEAD, THF, r.t.; 2. NH_2_NH_2_·H_2_O, EtOH, reflux, 82%; (ii) PMB-N=C=O, CH_3_CN, r.t., 92%; (iii) TMSOTf, CH_3_CN, 0 °C to r.t., 97%; (iv) Na_2_CrO_7_, H_2_SO_4_, acetone, r.t., 70%; (v) CAN, CH_3_CN/H_2_O, 100%; (vi) Mo(CO)_6_, CH_3_CN/H_2_O, 70%; (vii) CF_3_COOH/H_2_O (1:3), 0 °C, 100%.

Since the isomer bearing the nitrogen in the α-anomeric position is thermodynamically more stable than the desired β-isomer, the main challenge in the synthesis of hydantocidin is the control of the anomeric configuration. In an attempt to overcome this limitation, Chemla described an alternative synthesis of hydantocidin from protected psicofuranose **15** using as key step an oxygen-bridged intramolecular Vorbrüggen coupling of an intermediate *N*-hydroxyurea [39]. Initially, **15** was converted into the *p*-methoxybenzylurea **16**, which on treatment with a catalytic amount of trimethylsilyltriflate led to the isoxazolidine **17** (Scheme 3).

After oxidation of the free hydroxy group in **17** to the corresponding carboxylic acid and removal of the PMB protecting group, subsequent cyclization to the tricyclic isoxazolidine hydantocidin **18**, followed acidic hydrolysis finally afforded desired (+)-hydantocidin (**1**).

**Scheme 4:**
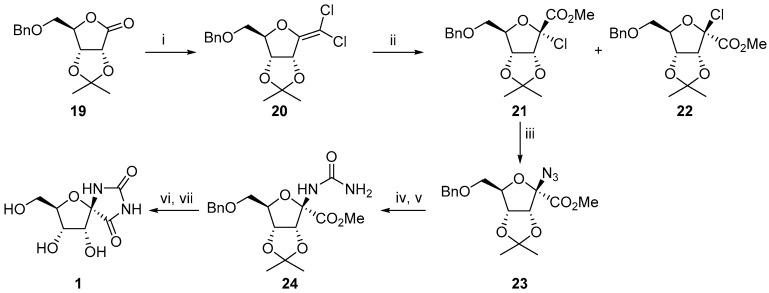
Shiozaki et al. route to (+)-hydantocidin (**1**). Reagents and conditions: (i) CBrCl_3_, (Me_2_N)_3_P, CH_2_Cl_2_, −78 °C to r.t., 16 h, 86%; (ii) m-CPBA, MeOH, CH_2_Cl_2_, r.t., 16 h, 54% (**21**) and 14% (**22**); (iii) NaN_3_, DMF, 24 °C, 16 h, 95%; (iv) 1. PPh_3_, THF, 24 °C, 2 h; 2. PMBNCO, THF, 24 °C, 16 h, two steps 90%; (v) 1. 1:20 aq 1 M HCl–THF, r.t., 15 min, quant. 2. CAN, 2:1 MeCN/water, r.t., 20 min, 97%; (vi) 0.2 M NH_3_ in MeOH, 27 °C, 4 h, 99%; (vii) 1. H_2_, Pd/C, EtOAc, 24 °C, 30 min; 2. 1:3 TFA/H_2_O, 0 °C, 2 h, quant.

In spite of the considerable synthetic work focused to the synthesis of hydantocidin, the problem of accessing multigram quantities remained unresolved. In 2002, Shiozaki reported a synthesis of hydantocidin from dichloroolefin **20**, easily available from protected d-ribonolactone **19** (Scheme 4) [40]. Oxidation of **20** with MCPBA afforded a 4:1 anomeric mixture of chlorosugars **21** and **22**. Conversion of **21** to urea **24** was easily accomplished via azide **23**. Finally, formation of the hydantoin ring, followed by hydrogenolysis and acidic hydrolysis yielded hyantocidin (**1**).

### 2.2. Modifications of Hydantocidin in the Sugar Ring

In order to elucidate the role of the sugar part of the hydantocidin molecule in the herbicidal activity, several hydantocidin diastereomers, deoxy derivatives, pyranose analogues and carbocyclic derivatives were synthesized.

#### 2.2.1. Furanoses

**Figure 2:**
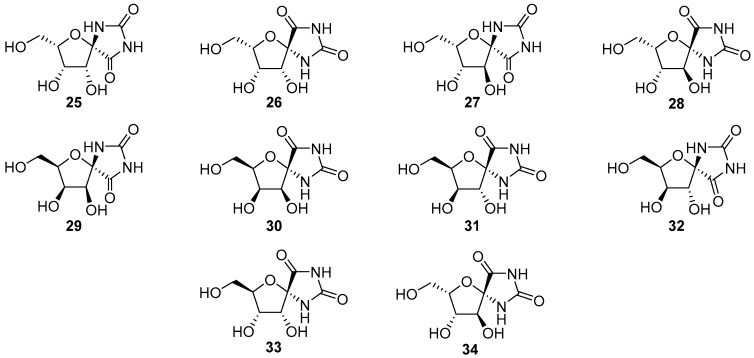
Examples of stereoisomers of hydantocidin.

The basic structure of this spironucleoside is comprised by a spiro-hydantoin ring at the anomeric carbon of a d-ribofuranose. That is a total of four stereocenters, which could play a fundamental role in the processes of recognition of the molecule at the active site of herbicidal action in the plant. All hydantocidin stereoisomers have been synthetized; however, sometimes the different isomers are prepared following the same synthetic sequence just varying the starting material. To avoid repetition, only the syntheses of several representative examples hydantocidin isomers are herein described (Figure 2).

**Scheme 5:**
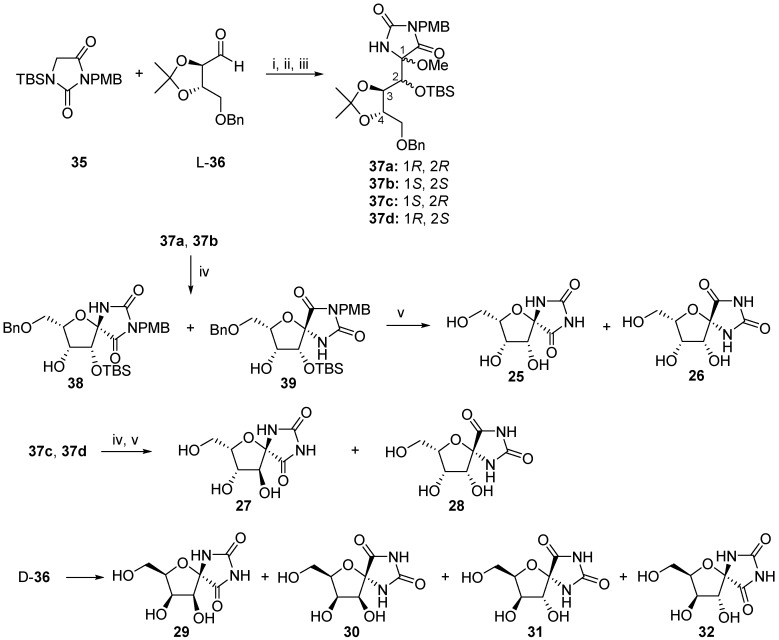
Mio and co-workers’ preparation of the diastereoisomers of (+)-hydantocidin (**1**). Reagents and conditions: (i) LiN(TMS)_2_, THF, −20 °C, 2 h, 76%; (ii) *t*-BuOK, MeOCOCl, THF, 0 °C, 30 min; (iii) aq K_2_CO_3_, MeOH, r.t., 1.5 h; (iv) *p*-TsOH, ethylene glycol, dichloroethane, 60 °C, from **37a**: 71% of **38** and 14% of **39**; from **37c**: 74% of **38** and 15% of **39**; (v) 1. TBAF, THF, 0 °C, 15 min, 78–85%; 2. CAN, 2:1 MeCN/water, r.t., 20 min, 78–86%; 3. H_2_, Pd/C, EtOAc, 24 °C, 30 min, 61–81%.

Once accomplished the synthesis of the natural product itself, Mio and co-workers reported the preparation of all the stereoisomers of hydantocidin [41]. For example, starting from the substituted hydantoin **35** an aldol condensation with tetrose l-**36** afforded condensate **37** as a mixture of four diastereomers (Scheme 5).

After chromatographic separation, isomers **37a** and **37c** were transformed into the same pair of two cyclized isomers **38** and **39**, which after removal of the protecting groups provided isomers **25** and **26**. Similarly, isomers **37b** and **37d** provided the other two isomers of the l-series, compounds **27** and **28**. In addition, when the same sequence of reactions was applied to aldehyde d-**36**, the four isomers of the d-series were obtained.

**Scheme 6:**
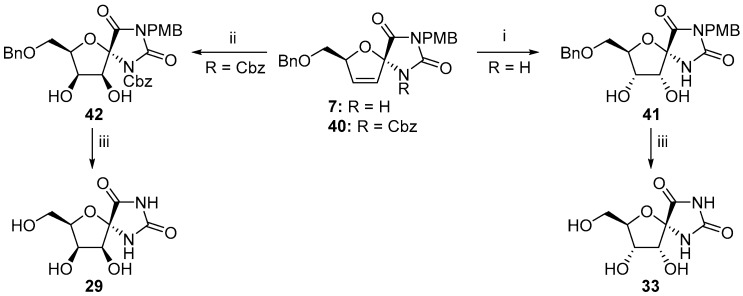
Mio et al. general synthetic route to the d-sugar diastereoisomer series. Reagents and conditions: (i) OsO_4_, *N*-methylmorpholine-*N*-oxide, acetone/H_2_O, r.t., 6 h, 76%; (ii) OsO_4_, *N*-methylmorpholine-*N*-oxide, acetone/H_2_O, 35 °C, 67 h, 53%; (iii) 1. CAN, 2:1 MeCN/H_2_O, r.t., 15 min, 50%; 2. H_2_, Pd/C, EtOAc, 55 °C, 6 h, 66%.

Based on the diastereoselective dihydroxylation of spiro-2,5-dihydrofuran systems, Mio and co-workers also reported a general synthetic route for the diastereoisomers of the series of d-sugars [42]. In this methodology, the selectivity is controlled by the choice of substituents at *N*-6 position (H or Cbz). Thus, catalytic osmium tetroxide oxidation of spiro-2,5-dihydrofurane **7** afforded a single isomer **41**, while oxidation of the *N*-benzyloxycarbonyl protected derivative **40** gave isomer **42** (Scheme 6). The *cis*-isomers **41** and **42** were then easily converted to hydantocidin stereoisomers **29** and **33**.

**Scheme 7:**
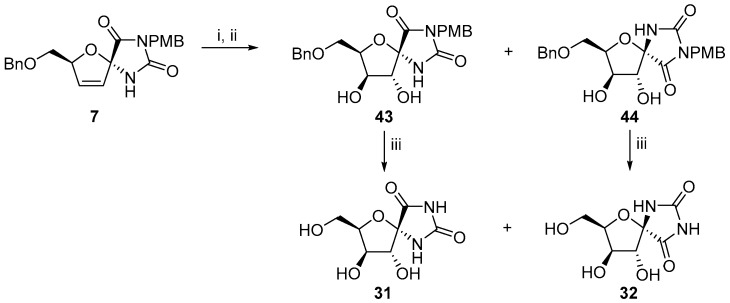
Mio et al. synthesis of the *trans* isomers. Reagents and conditions: (i) *m*-CPBA, dichloroethane, reflux, 4 h, 41%; (ii) 50% aq. H_2_SO_4_, DME, 50 °C, 7 h, 17% of **43** and 30% of **44**; (iii) 1. CAN, MeCN/H_2_O (2:1), r.t.; 2. H_2_, Pd/C, EtOAc, 55 °C, 21% of **31** and 27% of **32**.

For the synthesis of the *trans* isomers, the strategy involves the opening of the corresponding 3,4-epoxides in an acidic medium (Scheme 7). Epoxidation of **7** with *m*-chloroperbenzoic acid, followed by acidic ring-opening gave dihydroxy compounds **43** and **44**, as the rather drastic acidic conditions used for the epoxide opening resulted in the epimerization at the anomeric position. Interestingly, the ring opening occurred selectively on the C-3 position, although the factor involved in this regioselectivity are unknown. Removal of the protecting groups finally afforded the desired *trans* isomers **31** and **32**.

**Scheme 8:**
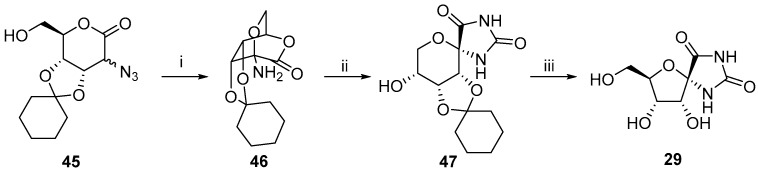
Fleet et al. short synthesis of 5-*epi*-hydantocidin (**29**). Reagents and conditions: (i) TPAP, morpholine-*N*-oxide, MeCN, r.t., 1 h, 60%; (ii) KOCN, AcOH, 60 °C, 1.5 h, 76%; (iii) 1. *tert*-BuOK, THF, r.t. 10 min, 61%; 2. aq. CF_3_COOH, r.t., 2 h, 98%.

Fleet et al. reported a short synthesis of 5-*epi*-hydantocidin **29** from a d-ribose-derived azidolactone [43]. Oxidation of azidonolactone **45** [44] with tetra-n-propylammonium perruthenate (TPAP) in the presence of morpholine-*N*-oxide gave a single product **46** (Scheme 8).

Treatment of **46** with potassium cyanate in acetic acid afforded the corresponding urea, which on reaction with potassium *tert*-butoxide and acidic hydrolysis gave epihydantocidin **29**.

**Figure 3:**
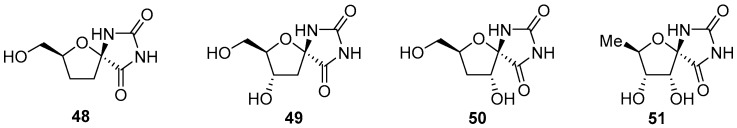
Deoxy analogues of hydantocidin.

In order to gain further insight into the role of the hydroxyl groups in the structure-herbicidal activity, a number of deoxy-derivatives of hydantocidin were also prepared (Figure 3).

**Scheme 9:**
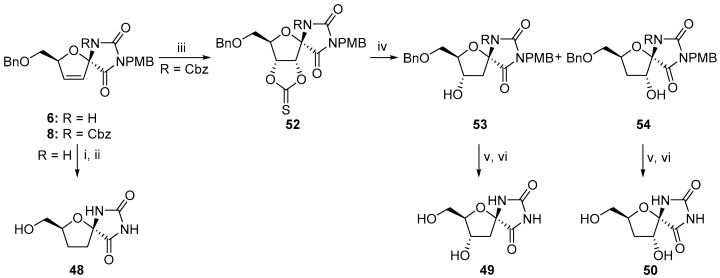
Synthesis of monodeoxyisomers **49** and **50**. Reagents and conditions: (i) CAN, 2:1 MeCN/water, r.t., 7 min, 86%; (ii) H_2_, Pd/C, EtOAc, 55 °C, 5 h, 65%; (iii) (imd)_2_CS, toluene, 100 °C, 1 h, 69%; (iv) 1. *n*-Bu_3_SnH, AIBN, toluene, r.t., 39% of **53** and 30% of **54**; (v) CAN, MeCN/H_2_O (2:1), r.t.; (vi) H_2_, Pd/C, EtOAc, 55 °C, 75% of **49** and 80% of **50**.

Starting from spiro-hydantoin derivative **6**, deprotection of the *p*-methoxybenzyl group followed by hydrogenation afforded dideoxy derivative **48** (Scheme 9) [45]. Mono-deoxyisomers **49** and **50** were synthesized from common intermediate **52**, which was derived from **8** via thiocarbonylation, radical reduction and deprotection.

**Scheme 10:**
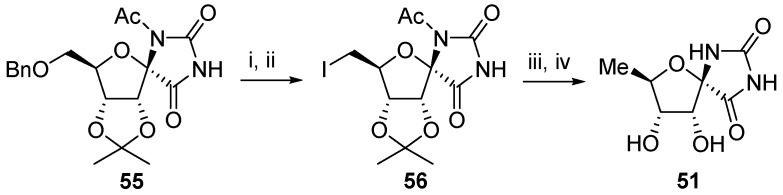
Synthesis of deoxyhydantocin **51.** Reagents and conditions: (i) H_2_, Pd/C, EtOAc, 55 °C, 78%; (ii) (PhO)_3_P^+^I^−^, DMF, r.t., 2 h, 90%; (iii) *n*-Bu_3_SnH, AIBN, toluene, r.t., 85%; (iv) 1. Dowex 50W (H^+^), MeOH/H_2_O, 70 °C, 1 h, 85%; 2. NH_2_NH_2_, MeOH, r.t., 30 min, 70%.

For the synthesis of deoxy-hydantocin **51**, compound **55** was chosen as starting material (Scheme 10). Debenzylation and iodination of the resulting hydroxy derivative yielded iodo-hydantocidin **56**. Removal of the iodine under radical conditions, followed by acidic hydrolysis of the isopropylidene protecting group finally afforded deoxy-hydantocidin **51**.

**Figure 4:**
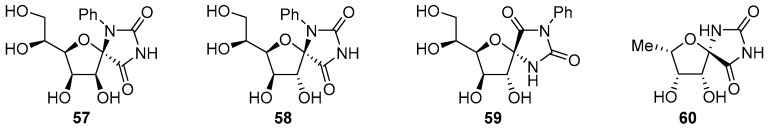
Hydantoins of pentoses and hexoses.

Even though the mode of herbicidal action of hydantocidin remained elusive at that time, Fleet and co-workers surmised that spirohydantoins of other sugars could also possess other interesting biological properties, so they initiated extensive investigations targeting diverse hydantoins of pentoses other than ribose and hexoses (Figure 4).

**Scheme 11:**
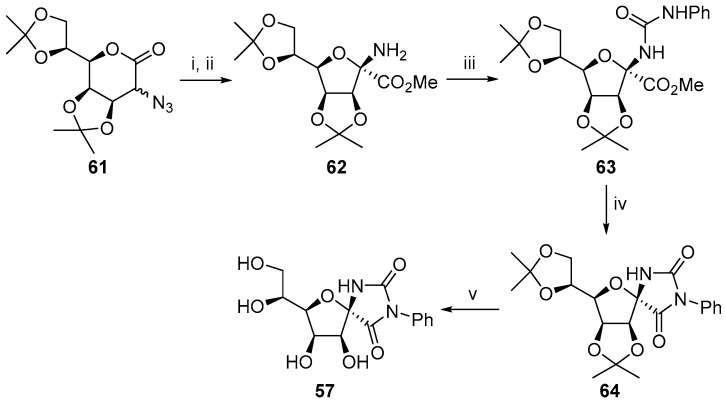
Hexose-derived hydantoin synthesis. Reagents and conditions: (i) H_2_, Pd black, EtOAc, r.t., 60%; (ii) Br_2_, NaOAc, MeOH then Et_3_N (iii) PhNCO, THF, r.t.; (iv) MeOH; (v) 1. 80% aq. AcOH, r.t. 2. 40% aq. CF_3_CO_2_H, r.t., 96%.

For example, Fleet’s group reported the first example of hexose-derived hydantoins, in a synthetic sequence using as key step an oxidative ring contraction of an α-amino-δ-lactone [46]. Hydrogenation of the azidolactone **61** gave the corresponding amine, which on oxidation with bromine in methanol in the presence of sodium acetate, followed by addition of triethylamine gave the amine ester **62** as the major product (Scheme 11). Reaction of **62** with phenyl isocyanate afforded the urea **63**, which on standing in methanol, spontaneously cyclised to the fully protected hydantoin **64**. Acidic hydrolysis of **64** gave the unprotected phenylhydantoin **57**.

**Scheme 12:**
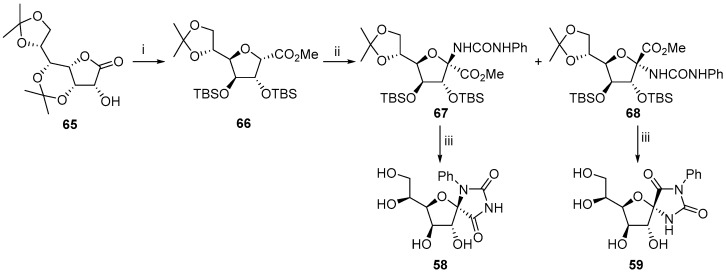
Glucose-derived hydantoin synthesis. Reagents and conditions: (i) 1. (CF_3_SO_2_)_2_O, Py, CH_2_Cl_2_, 77%; 2. H^+^, Me_2_CO, r.t., 80%; 3. Me_2_*^t^*BuSiCl, imidazole, DMF, 65 °C, 80%; (ii) 1. NBS, (PhCOO)_2_, CCl_4_, 80 °C; 2. NaN_3_, DMF, r.t., 37% and 34%; 3. H_2_, Pd, MeOH, r.t.; 4. PhNCO, THF, r.t., 36% of **67** and 63% of **68**; (iii) 1. *tert*-BuOK, DMF, 88% and 78%; 2. CF_3_COOH, dioxane, H_2_O, 73% of **58** and 75% of **59**.

For the synthesis of glucose-derived hydantoins, readily available glucoheptonolactone was used as starting material [47]. Ring contraction of glucoheptonolactone derivative **65**, followed by protection of the diol with *tert*-butyldimethylsilyl chloride, afforded the tetrahydrofuran **66** (Scheme 12). 

Radical bromination and subsequent reaction of the resulting bromides with sodium azide gave, after hydrogenation and reaction with phenyl isocyanate, ureas **67** and **68**. Potassium *tert*-butoxide-promoted cyclisation, followed by acidic hydrolysis, finally gave anomeric spirohydantoins **58** and **59**.

**Scheme 13:**
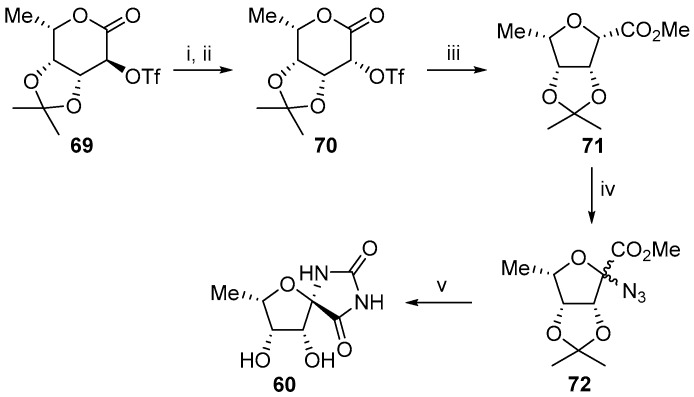
Fleet et al. synthesis of epimeric azidoesters. Reagents and conditions: (i) CF_3_COONa, DMF; (ii) Tf_2_O, pyridine, −30 °C, 84%; (iii) 1. 3% HCl in MeOH; 2. 2,2-dimethoxypropane, CSA, acetone, 97%; (iv) 1. NBS, CCl_4_, (PhCOO)_2_; 2. NaN_3_, DMF, r.t., 74%; (v) 1. H_2_, Pd/C, MeOH, 70%; 2. KOCN, AcOH, 74%; 3. 50% aq TFA, 94%.

As yet another contribution of Fleet’s group to the chemistry of sugar hydantoins, in 2006 these researchers reported the synthesis of lyxofuranose analogues of hydantocidin [48]. l-Fucose-derived triflate **69** [49] was transformed into inseparable mixture of epimeric azidoesters **72** (Scheme 13). From this mixture, lyxofuranose hydantoin **60** was obtained using the methodology established in the group for the construction of a spirohydantoin ring (formation and subsequent cyclization of an intermediate urea).

#### 2.2.2. Pyranoses

**Figure 5:**
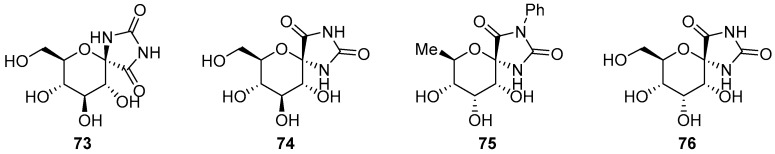
Pyranose derivatives of hydantocidin.

As stated in the introduction, Fleet and co-workers discovered a glucopyranose spirohydantoin which is the most active inhibitor of glycogen phosphorylase (GP) and therefore may be clinically useful for the treatment of diabetes mellitus. Inspired by the biological relevance of the glucopyranose hydantocidin, several pyranose analogues of hydantocidin were synthetized (Figure 5).

**Scheme 14:**
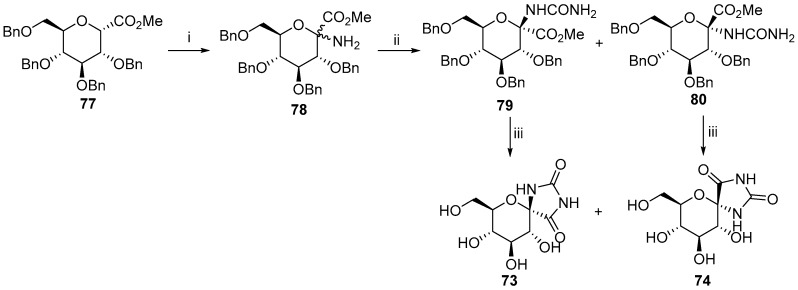
Glucopyranose hydantoin synthesis. Reagents and conditions: (i) 1. (Me_3_Si)_2_NLi then CBr_4,_ −0 °C; 2. NaN_3_, DMF, r.t., 66%; 3. H_2_, Pd black, MeCOOEt, r.t., 86%; (ii) KNCO, MeCOOH, 29% of **79** and 29% of **80** (iii) 1. *tert*-BuOK, THF; 2. H_2_, Pd black, EtOH, HCl, 81% of **73** and 73% of **74**.

Before pyranose analogues of hydantocidin were described, molecular modelling studies made a firm prediction that a glucopyranose analogue of hydantocidin would bind to and might strongly inhibit, glycogen phosphorylase. In order to confirm this hypothesis, Fleet and co-workers tackled the synthesis of the spirohydantoins of glucopyranose from the methyl ester **77** [50]. Treatment of **77** [51] with lithium bis(trimethylsilyl)amide and carbon tetrabromide gave an intermediate bromide, which was transformed into the ureas **79** and **80** via intermediate amines **78** (Scheme 14). Unlike in the case of the ribofuranosyl hydantoins, once the nitrogen of the quaternary anomeric centre has been acylated, there is no longer a kinetically easy pathway for the equilibration of the anomers to take place and **79** and **80** were separated by flash chromatography. Reaction of **79** with potassium *tert*-butoxide afforded, after removal of the protecting groups, glucopyranosyl hydantoin **73**. Following the same synthetic sequence, hydantoin **74** was obtained from **80**. As predicted by the molecular modeling studies, the spirohydantoin **73** is a potent inhibitor of glycogen phosphorylase, while **74** have a poor activity.

**Scheme 15:**
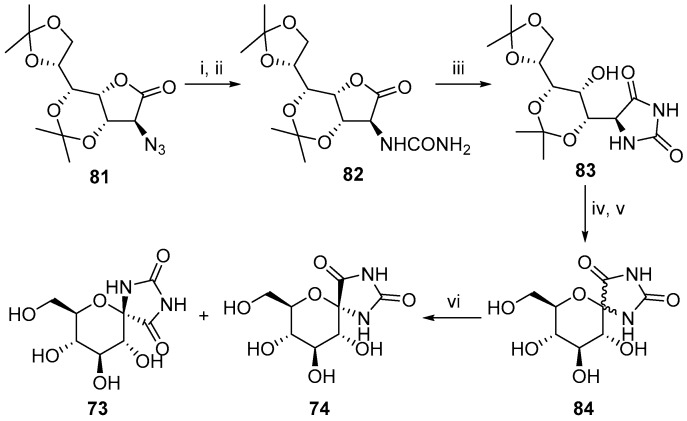
Reagents and conditions: (i) H_2_, Pd/C, THF, r.t., 100%; (ii) KOCN, AcOH, 20 min, r.t., 45%; (iii) AcOH, 1 h, 80 °C, 79%; (iv) AcOH/H_2_O (4:1), 1 h, 55 °C, 87%; (v) Br_2_, AcONa, MeOH; (vi) 1. PhCH(OMe)_2_, TsOH, DMF; 2. AcOH/H_2_O (4:1), 20 min, 75 °C; 3. Chromatographic separation; 4. H_2_, Pd black, MeOH, 19% of **73**, 9% of **74**.

This original synthesis of glucopyranosyl hydantoin **73** was lengthy and required a separation step, so was disadvantageous for a multi-gram preparation of the active compound. In view that furan isomers are thermodynamically more stable than their pyranose counterparts, all the attempts to isomerize spirofuran hydantoins to the pyranose isomers were abandoned. In turn, Fleet and co-workers reported yet another procedure for the synthesis of both anomeric glycopyranose hydantoins, this time from the cheap and readily available glucoheptonolactone [52]. (Scheme 15).

Starting from azide **81**, available on large scale from glucoheptonolactone, hydrogenation followed by treatment of the resulting amine with potassium cyanate in acetic acid, gave the urea **82**. After acidic hydrolysis to the open chain derivative **83**, bromine oxidation afforded the epimeric mixture of hydantoins **84**. Since it was not possible to separate the isomers directly, the anomeric mixture was converted to the corresponding benzylidene acetals, which were then separated by column chromatography and deprotected to finally yield both anomeric hydantoins **73** and **74**.

**Scheme 16:**
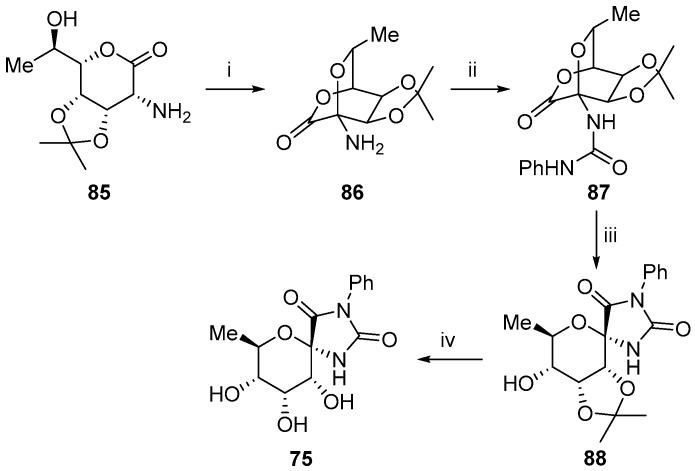
Reagents and conditions: (i) NBS, NaOAc, MeCN, r.t., 79%; (ii) PhNCO, pyridine, THF, r.t, 85%; (iii) MeOH, reflux, 76%; (iv) 50% aq. CF_3_COOH, r.t., 76%.

On account of the biological activity of spirohydantoin of glucopyranose **73** as a specific inhibitor of the glucosyl transferase glycogen phosphorylase, it was reasonable to assume that rhamnose hydantoins may interact with the active site of some rhamnose processing enzymes. Fleet and co-workers reported a route towards l-rhamnose hydantoins which includes as key step an ionic brominative oxidation of rhamnose derivative **85** [53] to bicyclic intermediate **86**, with both a nitrogen and a carbonyl function at the anomeric position (Scheme 16) [54]. Reaction of **86** with phenyl isocyanate and pyridine afforded the phenyl urea **87**, which spontaneously cyclised on refluxing methanol to afford the protected hydantoin **88**. Acidic hydrolysis finally gave the rhamnopyranose analogue of hydantocidin **75**.

**Scheme 17:**
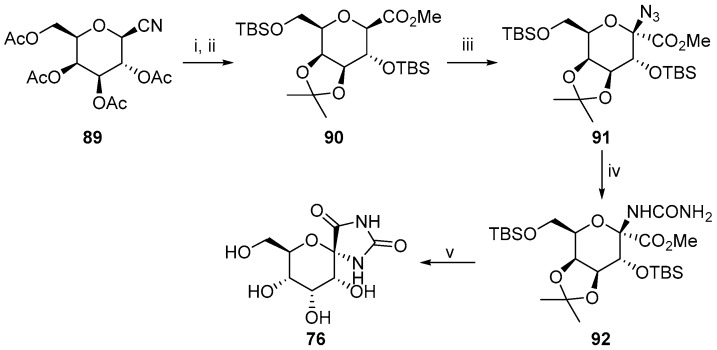
Reagents and conditions: (i) 1. MeOH, HCl; 2. Acetone, CSA, 60%; (ii) TBDMSOTf, NEt_3_, 100%; (iii) 1. NBS, (PhCO)_2_O, CCl_4_; 2. NaN_3_, DMF, 65%; (iv) 1. H_2_, Pd, MeOH; 2. KNCO, MeCOOH, 60%; (v) 1. KO*^t^*Bu, THF; 2. dioxane/H_2_O/CF_3_COOH (1:1:1), 86%.

In their continuous search for highly specific binding to enzymes or receptors involving carbohydrates, Fleet and co-workers also described the synthesis of a galactopyranose analogue of hydantocidin [55] (Scheme 17).

Starting from protected nitrile **89** [56], reaction with methanolic hydrogen chloride followed by isopropylidenation of the *cis*-1,2-diol and protection of the remaining hydroxy groups as *tert*-butyldimethylsilyl ethers afforded ester **90**. After radical bromination, intermediate bromide was transformed into urea **92**, which on *tert*-butoxide-induced cyclization and acidic hydrolysis afforded the desired galactopyranose analogue of hydantocidin **76**.

#### 2.2.3. Carbocycles

**Figure 6:**
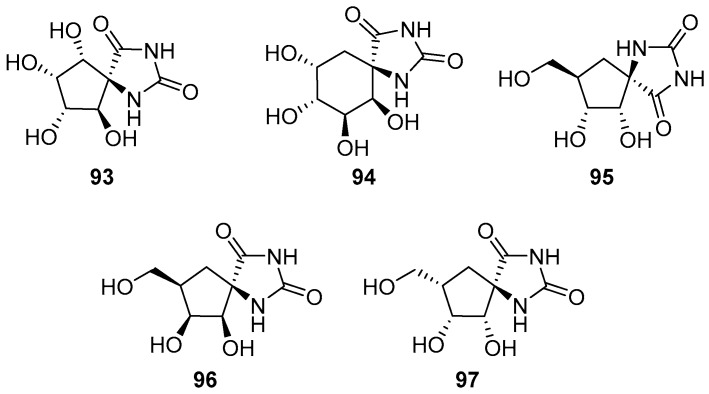
Carbocyclic analogues of hydantocidin.

Since (+)-hydantocidin possess a *N*,*O*-hemiacetal functionality at the anomeric position, it could be easily isomerized to the more thermodynamically stable 5-epimer. In order to avoid epimerization, a series of carbocyclic analogues were synthetized (Figure 6).

**Scheme 18:**
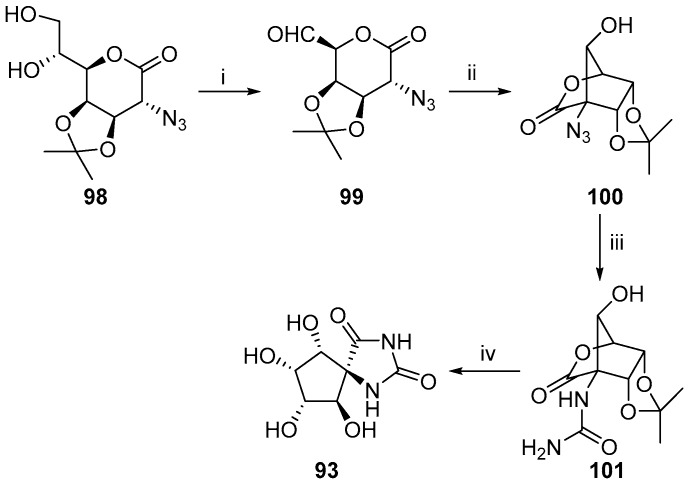
Reagents and conditions: (i) HIO_4_, THF, r.t., quant; (ii) KF, 18-crown-6, CH_3_CN, −6 °C, 58%; (iii) 1. H_2_, Pd black, aq. EtOH, r.t., 83%; 2. KOCN, AcOH, r.t., 90%; (vi) HCl, MeOH, r.t., quant.

The first synthesis of a carbocyclic hydantoin was reported by Fleet et al. in 1993 [57]. Intramolecular aldol reaction of aldehyde **99**, prepared from readily available azidodiol **98** [58], afforded bicyclic azidolactone **100** (Scheme 18). This lactone was converted into hydantoin **93** via the corresponding urea **101**.

**Scheme 19:**
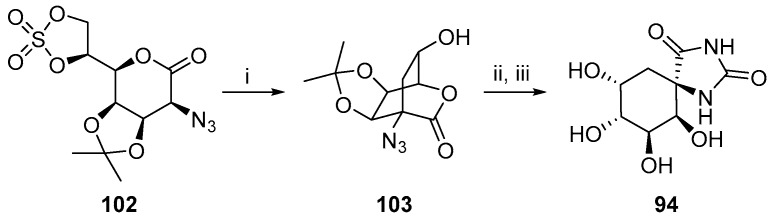
Reagents and conditions: (i) NaH, DMF then H^+^/H_2_O, 59%; (ii) 1. TFA/H_2_O, 70%; 2. H_2_, 10% Pd/C, EtOH, quant; (iii) 1. KOCN, AcOH, 87%; 2. HCl, MeOH, 88%.

The same researchers described the synthesis of a cyclohexane analogue of hydantocidin [59]. Thus, treatment of the azidosulphate **102** [60] with sodium hydride induced intramolecular cyclisation to azidolactone **103** (Scheme 19). From lactone **103**, the cyclohexane analogue of hydantocidin **94** was easily available following a synthetic sequence involving hydrogenation, formation of the urea and acidic hydrolysis.

**Scheme 20:**
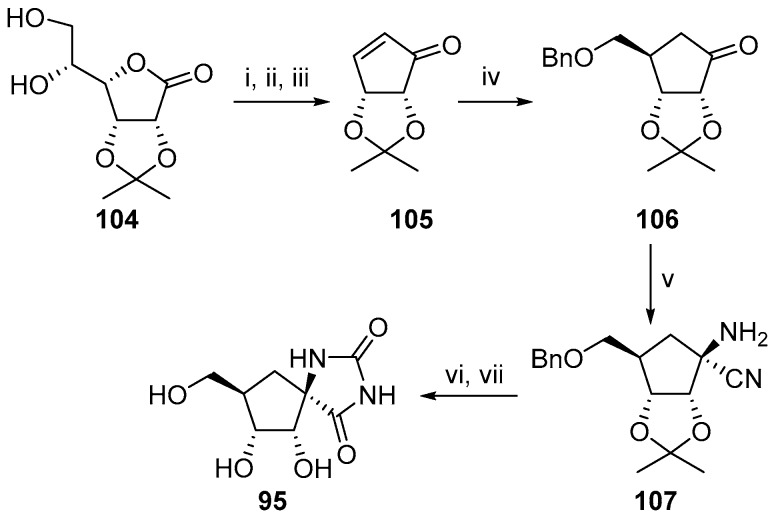
Reagents and conditions: (i) NaIO_4_, NaOH, H_2_O, r.t.; (ii) *p*-TsOH-Py, *i*-PrOH, 90 °C, 51%; (iii) MeP(O)(OMe)_2_, *n*-BuLi, THF, −8 °C to r.t., 51%; (iv) (BnOCH_2_)_2_CuBr 2Li Me_2_S, TMSCl, −8 °C to r.t., 99%; (v) KCN, NH_4_Cl; (vi) ClSO_2_NCO, CH_2_Cl_2_ then HCl 1 M, 100 °C, 64%; (vii) H_2_, Pd/C, MeOH, 55 °C, 98%.

Later in 1995, Sano et al. reported the synthesis of the carba-analogue of hydantocidin **95**, in which the oxygen atom of the d-ribose unit has been replaced by a methylene unit. After finding that the racemic carba-hydantocidin maintained the herbicidal activity [61], they focused on the synthesis of the optically active compound, which was prepared from easily available d-gulono-l,4-lactone [62]. Oxidative cleavage of gulonolactone **104** with sodium periodate, followed by formation of an intermediate isopropyl acetal and reaction with dimethyl methylphosphonate and *n*-butyl lithium, afforded cyclopentenone **105** (Scheme 20). Conjugate 1,4-addition of benzyl β-hydroxymethyl anion gave cyclopentanone **106** as a single isomer. Reaction of **106** with potassium cyanide, followed by treatment of the resulting aminonitrile **107** with chlorosulfonyl isocyanate afforded, after removal of the protecting groups, optically active carbocyclic analogue **95**.

**Scheme 21:**
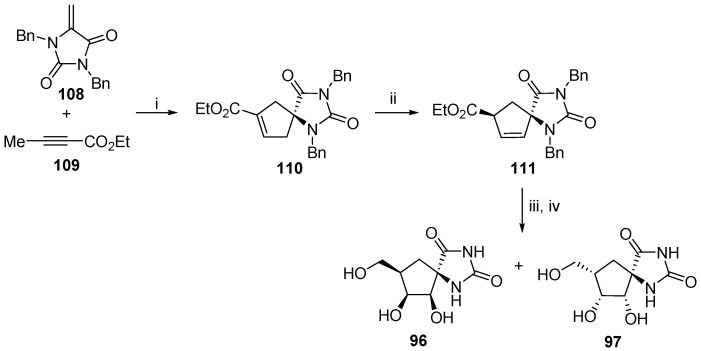
Reagents and conditions: (i) Bu_3_P, toluene, r.t; (ii) 1. KN(TMS)_2_, THF, −8 °C, 10 min; 2. HOAc, −8 °C to r.t., 99%; (iii) 1. 10% HCl, MeCN, 90 °C, 15 h, 99%; 2. BH_3_.DMS, THF, 0 °C, 6 h, 95%; 3. K_2_OsO_4_.2H_2_O, NMO, acetone/H_2_O (4:1), r.t., 5 days, 37%; 4. Ac_2_O, pyridine, MeCN, r.t., 10 h, 91%; (iv) 1. NBS, C_6_H_5_Cl, 125 °C, 14 h; 2. 10% HCl, THF, reflux, 4 h, 95–99%.

Most of the reported syntheses of hydantocidin and its analogues have revolved around the use of sugar derivatives to generate the desired stereochemistry of the hydroxyl groups in the furan ring. However, Pham and co-workers reported the preparation of carbocyclic hydantocidins **96** and **97** from ethyl 2-butynoate and *N*,*N*′-diprotected-5-methylenehydantoins using as key step a phosphine-catalysed [3 + 2]-cycloaddition to generate the spiro-heterocyclic system (Scheme 21) [63]. Thus, the cycloaddition reaction of the 5-methylenehydantoin **108** with the ylide that was generated in situ from the reaction of ethyl 2-butynoate **109** and tributylphosphine afforded ester **110**, which was then isomerized to ester **111** on treatment with potassium bistrimethylsilylamide. Acid catalysed hydrolysis of the ester group of **111**, followed by reduction and *cis*-dihydroxylation afforded, after removal of the protecting groups, carbocyclic hydantoins **96** and **97**.

### 2.3. Modifications of Hydantocidin in the Hydantoin Ring

**Figure 7:**
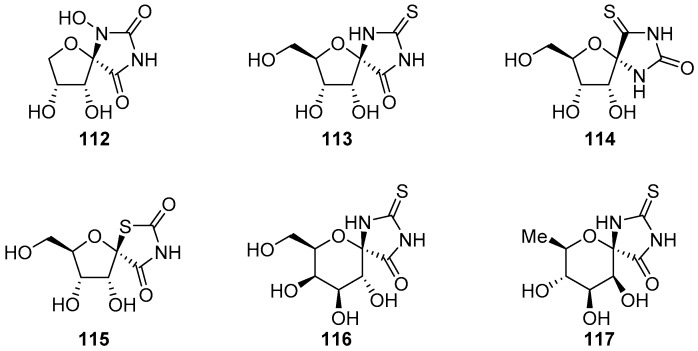
Analogues of hydantocidin modified in the hydantoin ring.

In order to shed some light on the functionalities required for the herbicidal action of hydantocidin, researchers also focused on the synthesis of structural analogues of hydantocidin resulting from modifications in the hydantoin ring of the parent molecule (Figure 7).

**Scheme 22:**
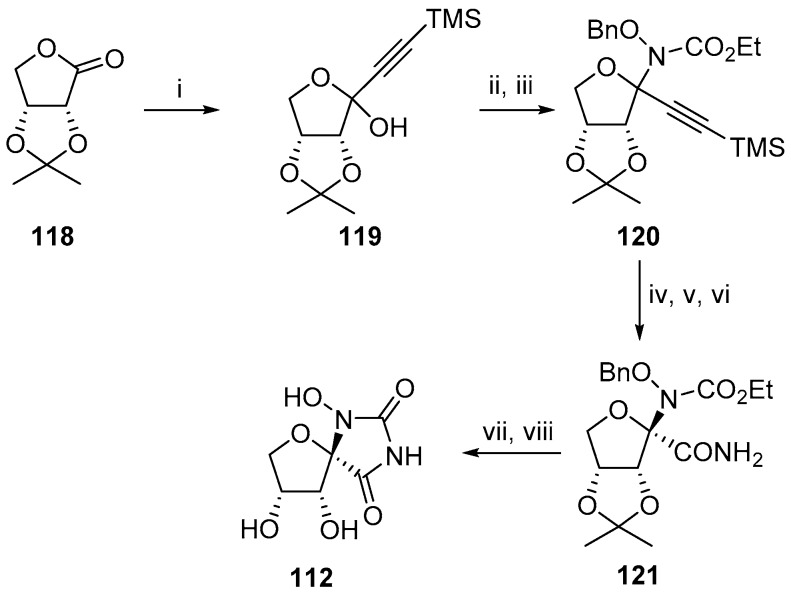
Reagents and conditions: (i) LiCCTMS; (ii) Ac_2_O, 80%; (iii) BnONHCO_2_Et, NaH, TfOTMS; (iv) 1. TBAF, THF; 2. H_2_, Pd/BaSO_4_, quinoline, 71%; (v) O_3_, CH_2_Cl_2_ then SMe_2_; (vi) 1. NaClO_2_, NaHPO_4_, 2-methyl-2-butene, *t*-BuOH, H_2_O; 2. PyBroP, NH_3_; (vii) TBAF, THF; (viii) 1. Dowex-H^+^; 2. H_2_, Pd/C, 83%.

For example, anticipating that the presence of a *N*-hydroxy group could provide a site for possible H-bonding and a polar environment on the β-face of the molecule, Hanessian and co-workers described the preparation of a *N*-hydroxyspirohydantoin **112** from readily available d-erythronolactone **118** (Scheme 22) [64]. Addition of lithium trimethylsilylacetylide gave **119**, which on acetylation and treatment with ethyl *N*-benzyloxycarbamate in the presence of sodium hydride and trimethylsilyl triflate, led to the formation of **120**. Removal of the TMS group and hydrogenation of the triple bond under Lindlar conditions gave the anomeric *C*-vinyl derivative, which on ozonolysis, oxidation of the resulting aldehyde and amidation with PyBroP afforded the corresponding anomeric amide **121**. Treatment of **121** with TBAF gave, after removal of the protecting groups, the intended hydantocidin analog **112**.

**Scheme 23:**
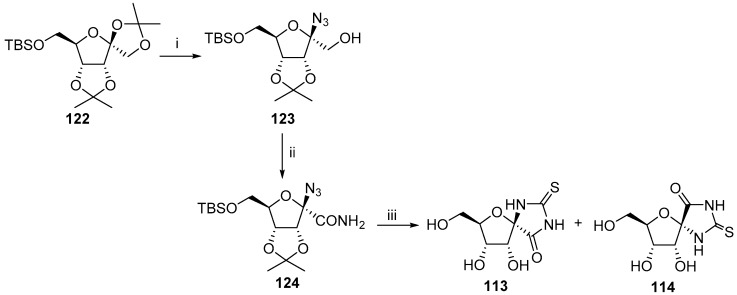
Reagents and conditions: (i) N_3_TMS, TfOTMS, CH_3_CN, 0 °C, 28%; (ii) 1. Swern; 2. NaClO_2_, NaHPO_4_, 2-methyl-2-butene, *t*-BuOH, H_2_O; 3. ClCO_2_Et, Et_3_N, NH_3_, 25%; (iii) Bu_3_P, CS_2_, 50 °C, 16% of **113** and 25% of **114**.

It was soon clear that drastic derivatization only led to diminished herbicidal activity, so attention of researchers turned to minimum modification without changing the basic structure. In this regard, Sano and co-workers described the first thiohydantoine derivative, in which the C7 carbonyl group was replaced with a thiocarbonyl group (compounds **113** and **114**) [65]. Starting with d-psicofuranose derivative **122** [38], selective cleavage of the exocyclic diol, followed by stereoselective introduction of an azide group in the anomeric position, afforded azide **123** (Scheme 23). Oxidation to the corresponding carboxylic acid, followed by coupling with ammonia, gave azide **124**. Spirothiohydantoin ring formation at the anomeric position was achieved by treatment of **124** with tri-*n*-butylphosphine and carbon disulfide affording, after acidic hydrolysis, spirothiohydantoin **113** along with its epimer **114**.

**Scheme 24:**
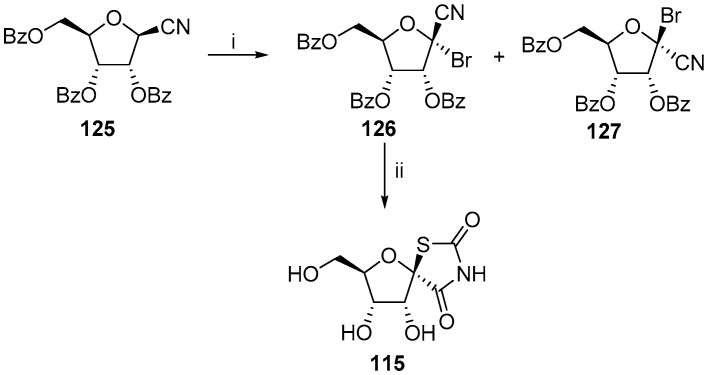
Reagents and conditions: (i) Br_2_, h⎨, CCl_4_, 93%; (ii) 1. H_2_NCSNH_2_, sulfolane; 2. 2 N HCl, 37%.

The observation that the introduction of the sulfur atom did not affect the herbicidal activity, sparkled the interest in thio analogues of hydantocidin. Thus, a number of analogues belonging to the family of thiohydantoins were described in the past few years. In an attempt to design analogues of hydantocidin which are more resistant to isomerisation, Lamberth and co-workers described the synthesis of a mimic of hydantocidin in which the hydantoin moiety was replaced by a thiazolidondione (compound **115**, Scheme 24) [66]. Photobromination of the readily available nitrile **125** [67] gave the bromo-ribosyl cyanide **126**, with on reaction thiourea and acidic hydrolysis afforded spiro-thiazolidindione **115**.

**Scheme 25:**
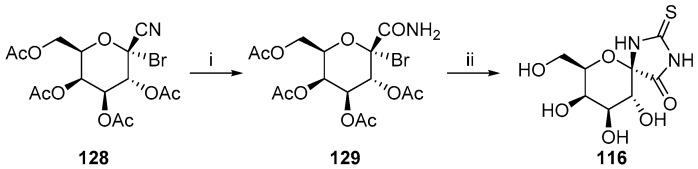
Reagents and conditions: (i) TiCl_4_, H_2_O, AcOH, r.t., 77%; (ii) 1. AgSCN, CH_3_NO_2_, 80 °C, 240%. 2. NaOMe, MeOH, r.t., 64%.

Somsák and co-workers developed an efficient general procedure for the short synthesis of thiohydantoins [68,69,70,71]. For example, Scheme 25 displays the synthesis of thiohydantoin **116** from d-galactopyranosyl cyanide **128** [72]. Thus, TiCl_4_ promoted addition of water to the nitrile moiety afforded formamide **129**, which on nucleophilic displacement of the bromide with silver thiocyanate followed by deacetylation furnished the pyranose thiohydantocidin **116**.

**Scheme 26:**
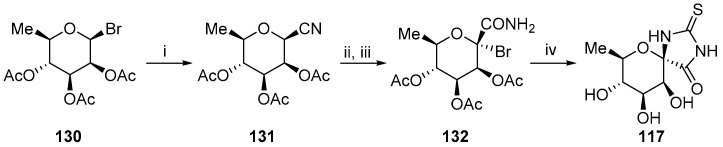
Reagents and conditions: (i) Hg(CN)_2_, AcOH, r.t., 48 h, 56%; (ii) HBr, CH_3_Cl, r.t., 3 h, 94%; (iii) Br_2_, CH_3_Cl, 80 °C, 7 h; (iv) NH_4_SCN, CH_3_NO_2_, r.t., 6 h, 63%.

Yet another synthesis of a thiohydantoin was reported in 2011 [73]. l-Rhamnopyranose bromide **130** (Scheme 26), obtained by a known procedure from l-rhamnopyranose [74], was treated with mercury(II) cyanide to give rhamnopyranose cyanide **131**. The partial hydrolysis of the nitrile moiety was accomplished on treatment with HBr in acetic acid to afford the corresponding formamide, which on photobromination gave derivative **132**. Reaction of **132** with ammonium thiocyanate in nitromethane in the presence of elemental sulfur finally gave spiro-thiohydantoin **117**.

## 3. Synthesis of Diketopiperazine Analogues

**Figure 8:**
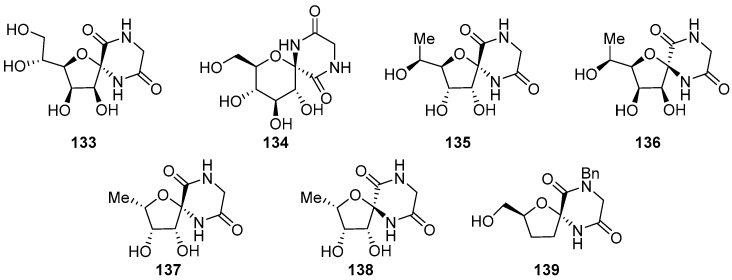
Analogues of hydantocidin containing a diketipiperazine ring.

As seen in the previous section, the bioactivity of hydantocidin has prompted extensive synthetic studies towards hydantocidin itself and a variety of its analogues. Additional preparative work has focused on the substitution of the hydantoin ring for other spiroheterocyclic subunits. In this regard, the potential of diketopiperazine analogues has not escaped attention. Diketopiperazines, both naturally occurring [75] and synthetic [76], are a class of bioactive peptides with a range of chemotherapeutic applications [77]. Given their biological relevance and in the search for novel mimics of hydantocidin, several syntheses of diketopiperazine analogues of hydantocidin were reported (Figure 8).

**Scheme 27:**
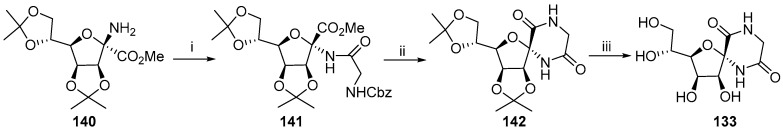
Reagents and conditions: (i) DCC, l-hydroxybenzotriazole, Cbz-Gly-OH, 72%; (ii) 1. H_2,_ Pd. MeOH, 91%; 2. *tert*-BuOK, THF, 89%; (iii) *tert*-BuOK, DMF, 100 °C, 12 h, 88% (vi) 1. AcOH, H_2_O; 2. CF_3_COOH, H_2_O, 80%.

In this regard, Fleet and co-workers extensively investigated the incorporation of a spirodiketopiperazine ring into the anomeric position of furanose and pyranose sugars [78,79]. Thus, on reaction with Cbz-glycine, amino ester **140** [80] isomerizes to the more nucleophilic amine intermediate before the actual coupling reaction, affording dipeptide **141** as the major product (Scheme 27). Removal of the Cbz-protecting group, followed by reaction with potassium *tert*-butoxide gave, after acidic hydrolysis, the desired spiro compound **133**. 

**Scheme 28:**
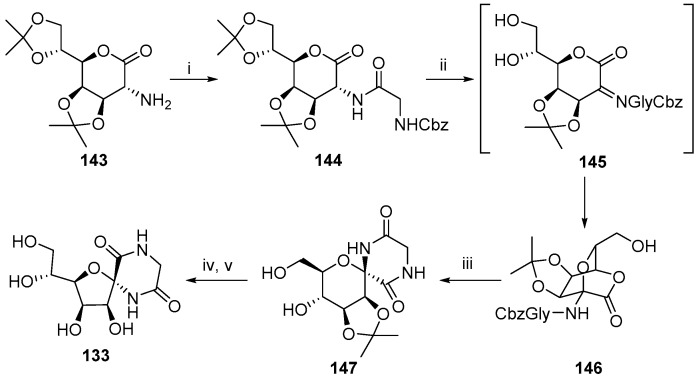
Reagents and conditions: (i) Cbz-Gly-OH, ClCOOEt, Py, MeCN/THF (1:1), 86%; (ii) 1. AcOH/H_2_O (1:1), 100%; 2. *N*-bromophthalimide, MeCOONa, 54%; (iii) H_2_, Pd. MeOH; (iv) CF_3_COOH, H_2_O; (v) *tert*-BuOK, DMF.

In an attempt to access the mannopyranose derivative, a slightly different strategy was used [81]. Coupling of the amine **143** with Cbz-glycine afforded derivative **144**. After selective removal of the exocyclic isopropylidene protecting group, oxidation with *N*-bromophthalimide afforded bicycle **146**. (Scheme 28). 

**Scheme 29:**
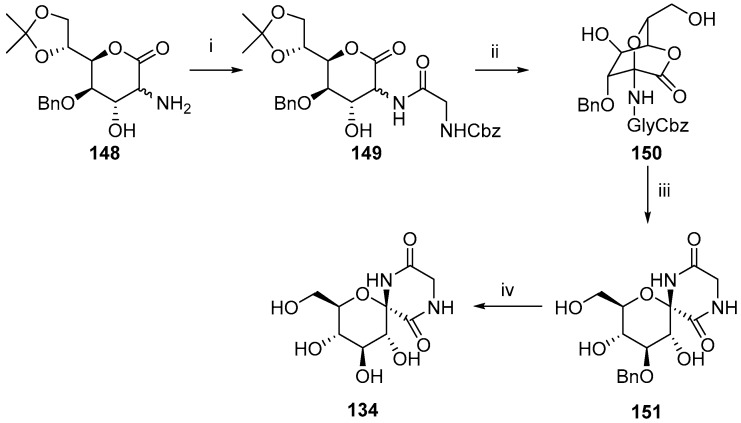
Reagents and conditions: (i) Cbz-Gly-OH, ClCOOEt, MeCN/THF (1:1), 88%; (ii) aq. AcOH with a trace of TFA, r.t., 82%; (iii) NBS, AcONa, CH_3_CN, 68%; (iv) Cyclohexene, 10% Pd/C, MeOH.

Removal of the benzyloxycarbonyl protecting group on hydrogenation was accompanied by spontaneously cyclisation of the resulting amine to give the spirodiketopiperazine **147**. However, acidic hydrolysis of **147**, followed by reaction with potassium *tert*-butoxide, gave mannofuranose diketopiperazine **133**, which is more stable than the corresponding mannopyranose derivative. In connection with their work aimed to discover efficient inhibitors of glycogen phosphorylase as possible therapeutic agents for the treatment of diabetes, Fleet and co-workers reported the synthesis of glucopyranosyl spirodiketopiperazine **134**, analogue of the bioactive glucopyranose hydantoin **73** [82]. Thus, coupling of lactone **148** with Cbz-glycine, followed by acidic hydrolysis and spontaneous cyclization, afforded the bicyclic lactone **150** (Scheme 29). Transfer hydrogenation of **150** gave a free amine which, on spontaneous cyclization yielded the spirodiketopiperazine **151**. Further transfer hydrogenation gave the required unprotected spiro compound **134**.

**Scheme 30:**
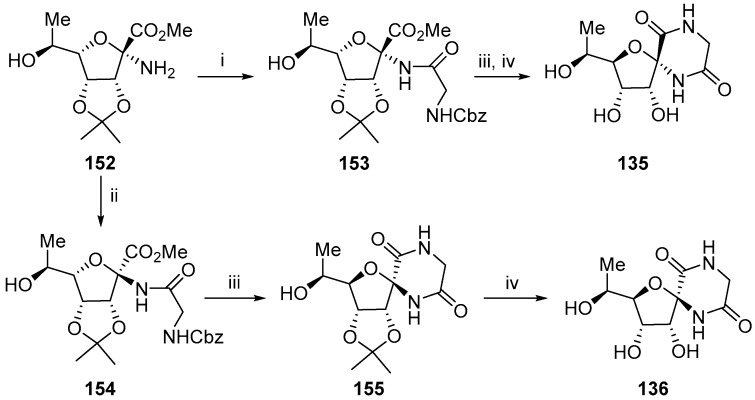
Reagents and conditions:(i) Cbz-Gly-OH, ClCOOEt, Et_3_N,THF; (ii) Cbz-GIy-OH, DCC, 1-hydroxybenzotriazole, DMF; (iii) H_2_, Pd black, EtOH; (iv) 50% aq. CF_3_COOH.

Fleet and co-workers also developed the synthesis of rhamnofuranose-derived diketopiperazines [54]. The aminoester **152** [83] was coupled with Cbz-glycine activated as a mixed anhydride with ethyl chloroformate, to give the dipeptide **153** in which the configuration at the anomeric centre has been retained (Scheme 30). Removal of the Cbz-protecting group gave an intermediate amine which spontaneously cyclised to the corresponding diketopiperazine, finally affording derivative **135** after acidic hydrolysis.

On the other hand, when Cbz-glycine was activated by DCC (*N*,*N*′-dicyclohexylcarbodiimide), the major product from coupling with the amine **152** was the dipeptide **154**. In the case of DCC activation, the carbonyl group is less electrophilic than is the case in activation by ethyl chloroformate. Thus, must equilibrate to the less stable, but more reactive, amine prior to acylation. Following a similar synthetic procedure as in the case of **153**, epimeric spirodiketopiperazine **136** was prepared from dipeptide **154**.

**Scheme 31:**
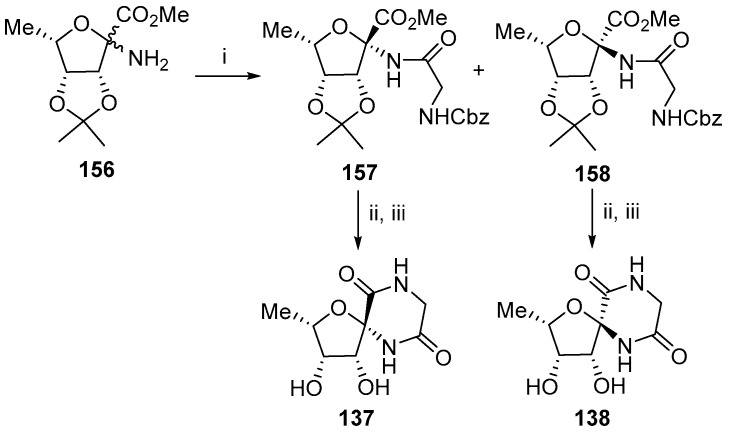
Reagents and conditions: (i) Cbz-Gly-OH, DCC, 1-hydroxybenzotriazole, dichloromethane, 73%; (ii) H2, Pd black, MeOH then *tert*-BuOK, THF, 44–69%; (iii) 50% aq TFA, 80%.

More recently, Feet et al. reported the synthesis of anomeric spirodiketopiperazines derived from 6-deoxy-l-lyxofuranose [48]. Using their established methodology for the construction of the spirodiketopiperazine ring, coupling of the mixture of epimeric aminoesters **156** with Cbz-glycine afforded epimeric dipeptides **157** and **158** (Scheme 31). Hydrogenolysis of the benzyloxycarbonyl protecting group in **157** gave the corresponding amine, which cyclized upon treatment with *t*-BuOK to afford, after acidic hydrolysis, the spirodiketopiperazine compound **137**. The same sequence was applied to dipeptide **158** to afford epimeric spirocyclic sugar **138**.

**Scheme 32:**
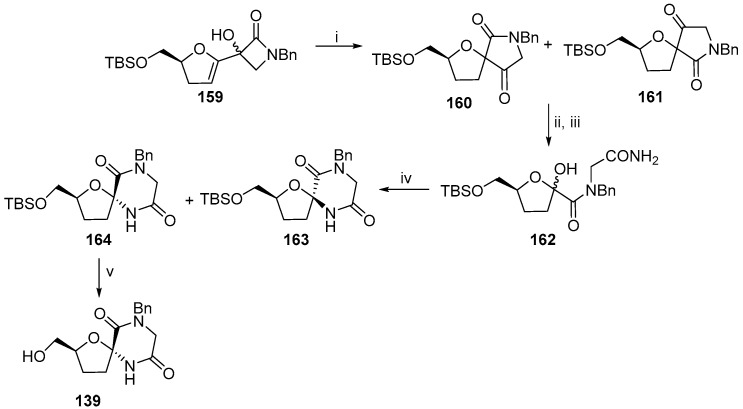
Reagents and conditions: (i) PPTS, DCM; (ii) *m-*CPBA, NaHCO_3_, DCM, 93%; (iii) NH_3_, MeOH, 100%; (iv) PPTS, benzene, 4 Å molecular sieves; (v) TBAF, THF, 100%.

After the pioneering work of Fleet’s group, other synthetic procedures have been developed for the preparation of different anomeric spirodiketopiperazines. For example, 2,3-dideoxy derivative **139** was synthesized using a procedure featuring an acid-catalyzed rearrangement of a 3-hydroxy β-lactam and the ammonolysis of a spiro keto lactone (Scheme 32) [84]. Starting from hydroxyazetidinone **159**, isomerization with PPTS gave derivatives **160** and **161**. Baeyer-Villiger oxidation the spiro system, followed by ammonolysis, yielded the lactol amide **162** as a 1:1 mixture of epimers. Ring closure and desilylation finally afforded diketopiperazine **139**.

## 4. Synthesis of Barbiturate Analogues

**Scheme 33:**
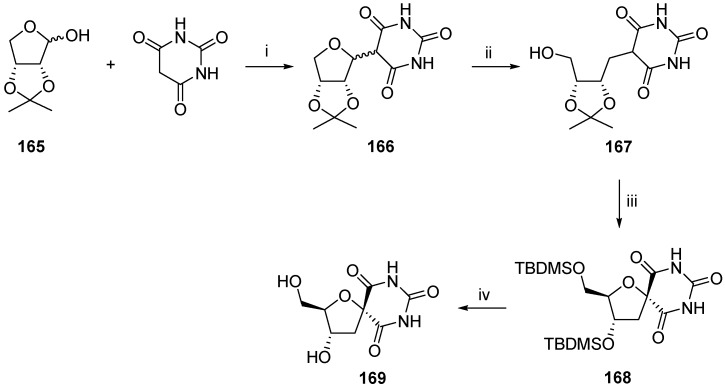
Reagents and conditions: (i) barbituric acid, Na_2_CO_3_, H_2_O, 59%; (ii) H_2_, Pd-C, MeOH, 91%; (iii) Br_2_, TBDMSCl, imidazole, DMF, 63%; (iv) TMSCl, MeOH, 95%.

A relevant problem with hydantoins and diketopiperazines is their lack of stability, mainly due to facile anomeric epimerization in basic media. To avoid epimerization around C-1′, hydantoin or diketopiperazine rings can be replaced by a barbiturate ring. Like the hydantoin ring, the barbiturate ring system possesses thymine-like hydrogen bonding capacity against adenine derivatives [85] and is found in many pharmaceutically relevant molecules [86]. In 2002, Renard and collaborators reported the synthesis of a spiro-barbituric deoxyribofuranose and its carbocyclic analogue from carbohydrate derivatives [87]. Erythrolactol **165** was condensed with barbituric acid in the presence of sodium carbonate to give the erythrosyl barbituric acid derivative **166** (Scheme 33). After hydrogenolysis, bromination of intermediate alcohol **167** in the presence *tert*-butyldimethylsilyl chloride afforded derivative **168**. Silyl deprotection finally gave the desired spiro-barbituric deoxyribofuranose **169**.

**Scheme 34:**
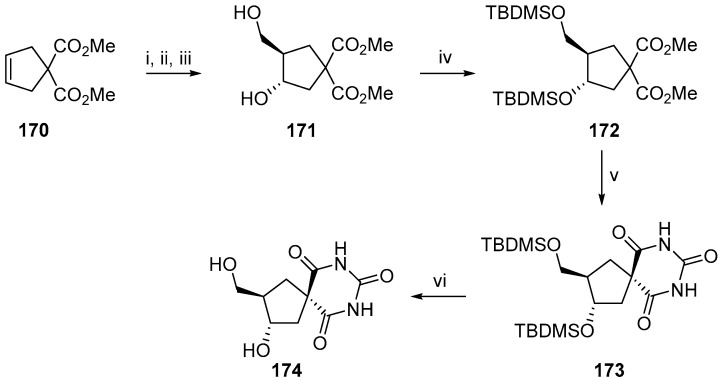
Reagents and conditions: (i) (CH_2_O)_n_, AcOH, H_2_SO_4_, 60 °C, 24 h; (ii) TMCl, MeOH; (iii) resolution, 60%; (iv) TBDMSCl, imidazole, DMF, 90%; (v) *t*-BuOK, urea, 68%; (vi) TMSCl, MeOH, 99%.

In order to enhance the stability of derivative **169**, the synthesis of a carba-sugar analogue was also described. Prins reaction of cyclopentene diester **170**, followed by pancreatin-catalyzed resolution (25%, *ee* > 98%) of the resulting racemic diol, afforded the optically pure diol **171** (Scheme 34). Silylation and condensation with urea afforded the spiro-barbituric acid **173**, which on deprotection of the silyl groups gave the desired carbocyclic analogue **174**.

## 5. Synthesis of Miscellaneous Spiroheterocyclic Units

**Figure 9:**
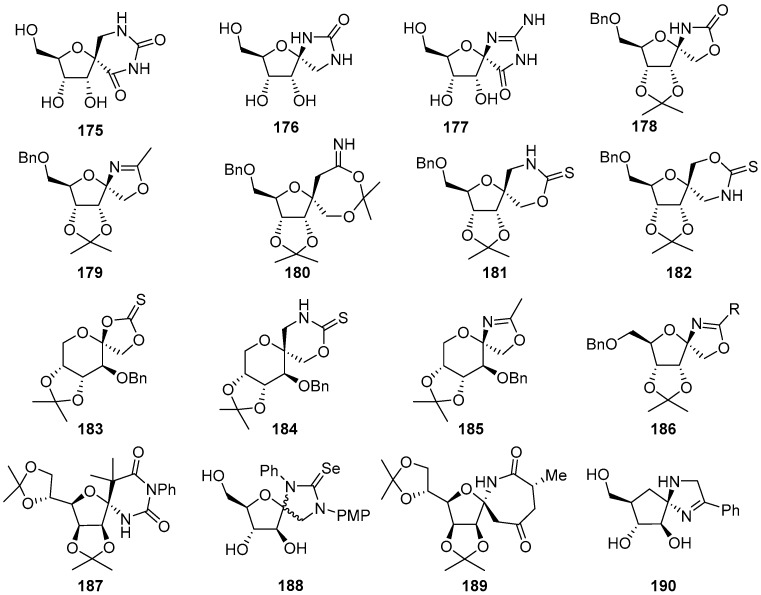
Hydantocidin analogues with miscellaneous spiroheterocyclic rings.

**Scheme 35:**
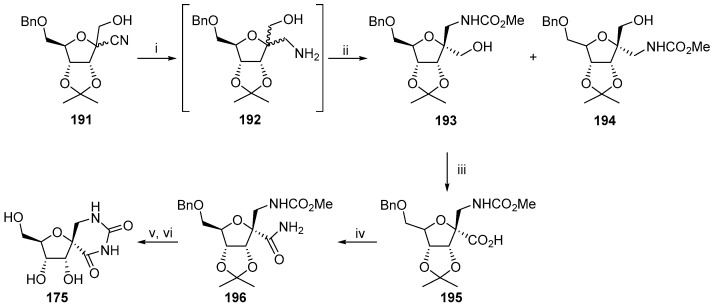
Reagents and conditions: (i) LiAlH_4_, THF, r.t., 0 °C, 30 min; (ii) ClCO_2_Me, CH_2_Cl_2_, NEt_3_, 0 °C, 20 min, 63% of **193** and 15% of **194**; (iii) 1. Swern; 2. NaClO_2_, MeOH, r.t., −0 °C, 20 min, 86% (iv) ClCO_2_Et, then NH_3_, 0 °C, 91%; (v) NaN(TMS)_2_, *n*-Bu_4_NF, THF, r.t., 86%; (vi) 1. Dowex, 50 W; 2. H_2_, Pd-C, r.t., 100%.

On addition to diketopiperazines and barbiturates, several other hydantocidin analogues with very diverse spiroheterocyclic rings were synthetized, as depicted in Figure 9. For example, in order to study the direction of the hydrogen bonding of the hydantoin, a spirodihydrouracil analogue of (+)-hydantocidin was developed [88]. Starting from mixture on nitriles **191**, reduction with lithium aluminium hydride afforded aminoalcohol **192**, which was *N*-carbonylated to give carbamates **193** and **194** (Scheme 35). Oxidation of the alcohol **193** to the corresponding carboxylic acid followed by condensation with ammonia provided amide **196**. The base-promoted intramolecular cyclization of **196** gave, after removal of the protecting groups, the spirodihydrouracil **175**.

**Scheme 36:**
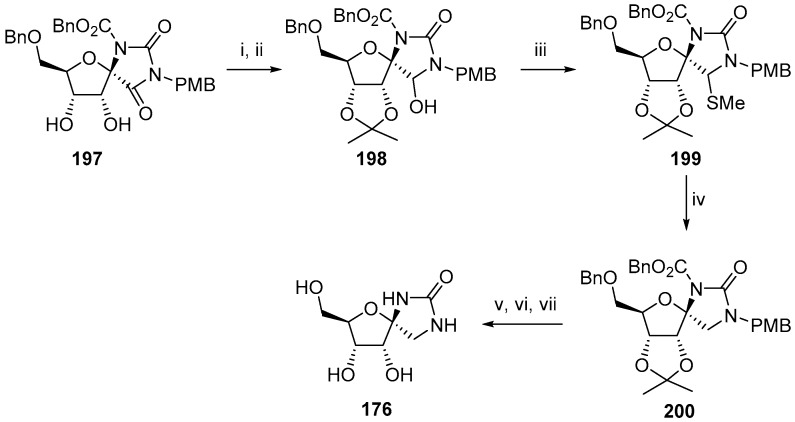
Reagents and conditions: (i) 2,2-Dimethoxypropane, *p*-TsOH·H_2_O, r.t., 50 min, 83%; (ii) NaBH_4_, MeOH, r.t., 40 min, 91%; (iii) *n*-Bu_3_P, MeSSMe, CH_2_Cl_2_, r.t., 28 h, 50%; (iv) *n*-Bu_3_SnH, AIBN, toluene, 100 °C, 1 h, 75%; (v) CAN, MeCN/H_2_O, r.t., 20 min, 51% (vi) *p*-TsOH·H_2_O, MeOH, r.t., 28 h, 79%; (vii) H_2_, Pd-C, 55 °C, 5 h, 45%.

Sano et al. prepared several hydantocidin analogues including modifications on the carbonyl groups of the hydantoin ring [89]. For example, spiroimidazolidinone **176** was synthesized using a demethyldesulfurization as key step (Scheme 36). Thus, compound **197** [36] was converted to the mixture of epimeric thioacetals **199**, which on radical demethylsulfurization afforded compound **200**. The final 3 steps consisted of the removal of the protecting groups, to finally afford analogue **176**.

**Scheme 37:**
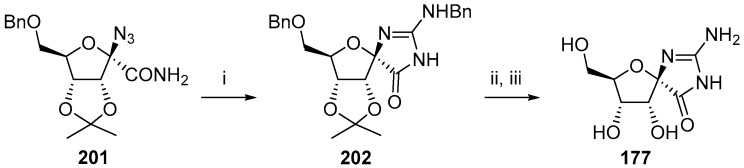
Reagents and conditions: (i) BnNCO, *n*-Bu_3_P, THF, r.t., 2 h, 98%; (ii) Dowex 50W (H^+^), MeOH/H_2_O, 80 °C, 1.5 h, 20%; (iii) H_2_, Pd-C, 55 °C, 5 h, 27%.

The synthesis of a spiroimidazolinone analogue of hydantocidin was also described (Scheme 37). Treatment of azido amide **201** with benzyl isocyanate and tri-*n*-butylphosphine afforded the spiro compound **202**, which on acid hydrolysis and hydrogenolysis gave desired spiroimidazolinone **177**.

**Scheme 38:**
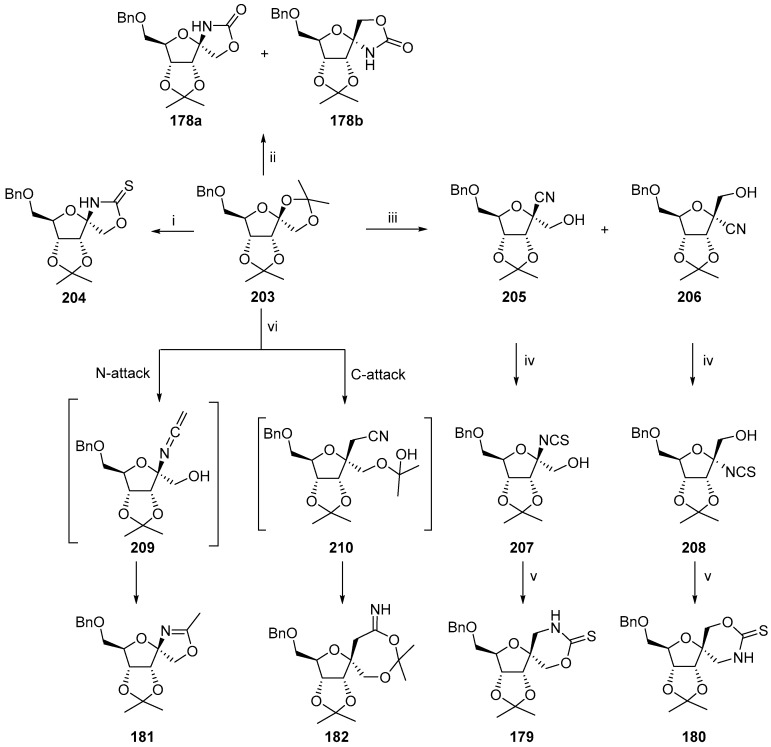
Reagents and conditions: (i) TMSNCS, TMSOTf, −20 °C, 1 h, 10%; (ii) TMSNCO, TMSOTf, −20 °C, 2 h, 22% (**178a**) and 5% (**178b**); (iii) Ref [74]; (iv) 1. LiAlH_4_, Et_2_O, 0 °C to r.t., 2 h; 2. Cl_2_CS, r.t., 6 h, 39% (**205**) and 36% (**206**); (v) NEt_3_, 80 °C, 40 min, 85% (**179**) and 93% (**180**); (vi) TMSCH_2_CN, TMSOTf, −20 °C, 23% (**181**) and 5% (**182**).

Gasch and collaborators reported the stereoselective synthesis of a wide range of pyranoid and furanoid spiroheterocyclic analogues of hydantocidin [90,91]. Thus, reaction of psicofuranose spiroketal **203** with trimethylsilyl isothiocyanate in the presence of trimethylsilyl triflate provided the corresponding *O*-protected thioxo-oxazolidine **204** (Scheme 38). The *N*-glycosylation of **203** with trimethylsilyl isothiocyanate in the presence of a Lewis acid afforded the mixture of oxazolidines **178a** and **178b**. For the synthesis of spiro-*C*-glycosides, spiroketal **203** was transformed into psicofuranosyl cyanides **205** and **206** according to Sano’s procedure [88]. Reduction of psicofuranosyl cyanide **205** with lithium aluminium hydride, followed by treatment with thiophosgene, afforded isothiocyanate **207**, which on treatment with triethylamine afforded the intramolecular cycloadduct **179**. Similarly, compound **180** was obtained from **206**. The reaction of **203** with (trimethylsilyl)acetonitrile afforded two products of the nucleophylic attack on the anomeric carbon, via either the nitrogen atom (*N*-attack) or the methylenic carbon (*C*-attack). The *N*-attack forms a heterocumulene intermediate **209**, whereas the *C*-attack produces intermediate nitrile **210**; both intermediates undergo intramolecular cyclization to finally afford **181** and **182**.

**Scheme 39:**
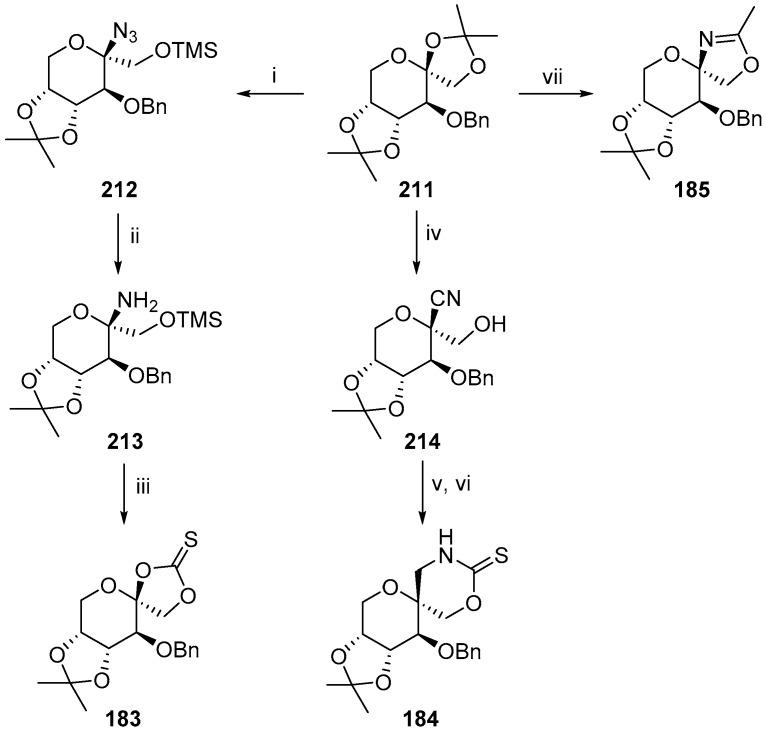
Reagents and conditions: (i) 1. TMSN_3_, 0 °C, 5 min; 2. TMSOTf, 0 °C, 5 min, 85%; (ii) H_2_/Pd-C, r.t., 2 h, 90%; (iii) 1. Bu_4_NF.3H_2_O, r.t., 1 h; 2. Im_2_CS, r.t., 3 h, 83%; (iv) 1. TMSCN, −0 °C, 5 min; 2. TMSOTf, 0 °C, 5 min; 3. Bu_4_NF.3H_2_O, r.t., 8 h, 55%; (v) a. LiAlH_4_, 0 °C, 30 min; b. Im_2_CS; (vi) Et_3_N, r.t., 10 h, 80%; (vii) TMSCH_2_CN, TMSOTf, −20 °C, 1 h, 75%.

Additional work allowed the preparation of other 6 + 5, 6 + 6 spironucleosides and spiro-*C*-glycosides from spiroketal derivative **211** (Scheme 39). *N*-glycosylation of **211** with trimethyl azide afforded **212**, as a 9:1 anomeric mixture. Pd-catalyzed hydrogenation of **212**, followed by treatment of the intermediate amine **213** with TBAF and thiocarbonyldiimidazole afforded spironucleoside **183**. *C*-glycosylation of **211** with trimethylcyanide in the presence of trimethylsilyltriflate, followed by desilylation with TBAF gave the fructopyranolsyl cyanide **214**. Reduction with lithium aluminium hydride, followed by reaction with thiocarbonyl diimidazole and triethylamine-promoted intramolecular cyclization, provided compound **184**. Finally, reaction of **211** with trimethylsilylacetonitrile in the presence of trimethylsilyltriflate afforded derivative **185**.

**Scheme 40:**
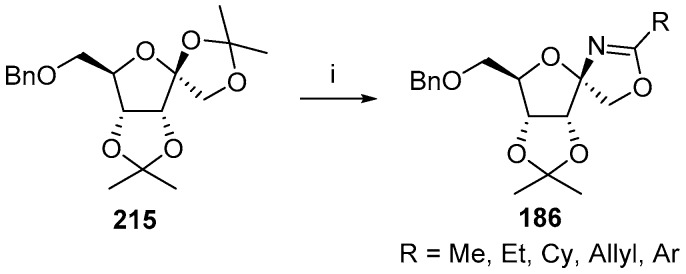
Reagents and conditions: (i) TMSOTf, R-CN, toluene, 0 °C to r.t., 1 h, 44–72%.

Also related to spironucleosides are the recently described spiro-oxazoline furanosides **186** [92] Their synthesis was achieved using a TMSOTf-promoted nucleophilic addition of electron-rich nitriles to the oxacarbenium ion intermediate of reaction of protected psicofuranose derivative **215** (Scheme 40). In addition to their pharmacological interest, spiro-fused carbohydrate oxazoline derivatives have potential applications in asymmetric catalysis [93].

**Scheme 41:**
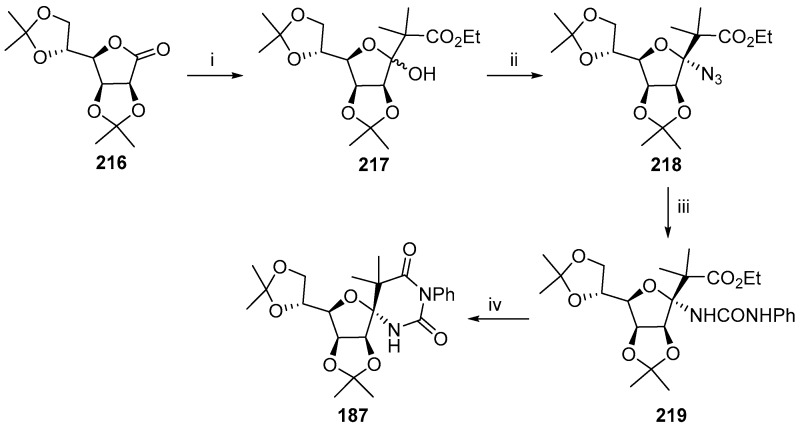
Reagents and conditions: (i) In, BrC(CH_3_)_2_CO_2_Et, THF, US, r.t; (ii) 1. Ac_2_O, Et_3_N, CH_2_Cl_2_, r.t., 14 h; 2. TMSN3, TMSOTf, powdered MS, CH_2_Cl_2_, r.t., 14 h, 71%; (iii) 1. Pd/C, MeOH, r.t., 14 h; 2. PhCNO, toluene, r.t., 3 h, 85%; (iv) NaOMe, r.t., 14 h, quant.

As a variation of the spirodiketopiperazine skeleton, the spiro-derivative **187** was recently prepared from carbohydrate lactones in a route involving *N*-glycosylation of ulosonic acid esters [94]. Thus, an indium-mediated Reformatsky reaction of mannonolactone diacetonide **216** with ethyl α-bromoisobutyrate gave ulosonate **217** (Scheme 41). *N*-glycosylation of compound **217** was achieved by acetylation followed by reaction with trimethylsilyl azide in the presence of trimethylsilyl triflate, affording azide **218** diastereoselectively. Catalytic hydrogenation and treatment of the resulting anomeric mixture of amino esters with phenyl isocyanate afforded derivative **219** as a single anomer. Basic treatment of urea **219** easily gave the corresponding diketopiperazine **187** without any equilibration of anomers taking place.

**Scheme 42:**
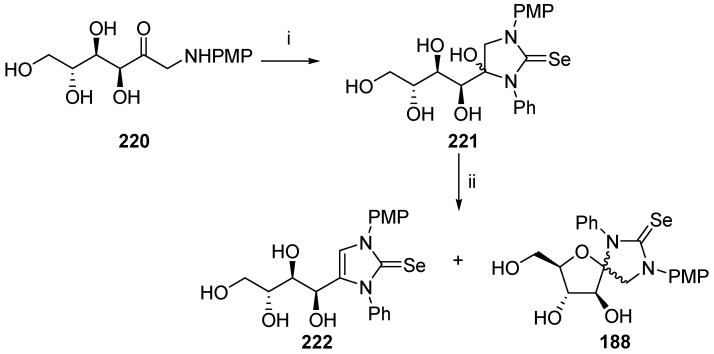
Reagents and conditions: (i) PhNCSe, DMF, r.t., 72 h, 98%; (ii) AcOH, EtOH/H_2_O (2:1), reflux, 1 h, 39% of **222** and 60% of **188**.

Maza et al. recently reported the first selenium-containing (+)-hydantocidin analogues [95]. Starting from *N*-arylfructosamine **220**, reaction with phenyl isoselenocyanate afforded the corresponding imidazolidine-2-selone **221**, which underwent dehydration under weak acidic conditions to give spiranic derivative **188** as a major compound (Scheme 42).

**Scheme 43:**
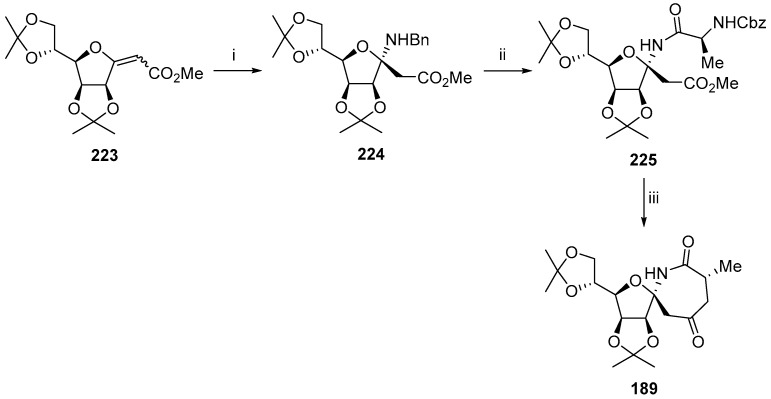
Reagents and conditions: (i) Neat BnNH_2_, 48 h, 91%; (ii) 1. H_2_/1 atm, 10% Pd–C, EtOAc; 2. Cbz-Ala-OH, PyBOP, Et_3_N, DMF, r.t., 92%; (iii) 1. H_2_/1 atm, 10% Pd–C, EtOAc; 2. K_2_CO_3_, MeOH/H_2_O 10:1, r.t., 48 h; 3. H_2_/1 atm, 10% Pd–C, EtOH/EtOAc 1.5:1; 4. DPPA, Et_3_N, DMF, 0 °C to r.t., 14 h, 47%.

Taillefumier and co-workers reported the first synthesis of 1,4-diazepine 2,5-dione anomeric spirosugars, which can be regarded as the first members of a new class of spironucleosides [96]. Michael addition of benzylamine to glycal **223** [97], followed by hydrogenation and coupling of the resulting free amine with Cbz-Ala-OH, afforded dipeptide **225**. After protecting group removal, base cyclization of dipeptide **225** on high dilution gave diazepine **189** (Scheme 43).

**Scheme 44:**
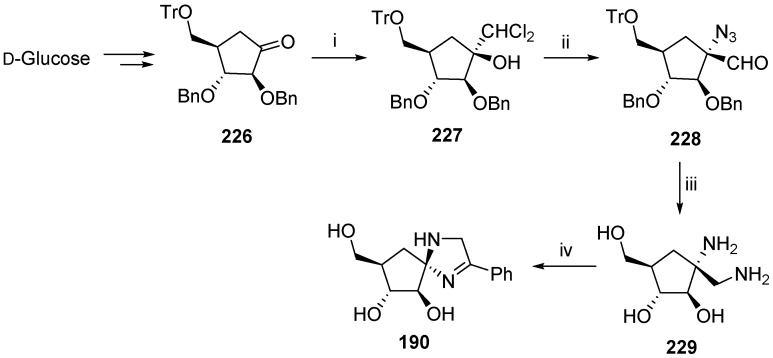
Reagents and conditions: (i) *i*-Pr_2_NH, *n*-BuLi, CH_2_Cl_2_, THF, 67%; (ii) NaN_3_, 15-crown-5, Me2SO, 79%; (iii) 1. BnNH_2_, NaBH_2_CN, AcOH, TFA, 86%; 2. H_2_, Pd(OH)_2_–C, HCl, MeOH, THF; (iv) 1. (Boc)_2_O, Et_3_N, MeOH, H_2_O; 2. TFA, CH_2_Cl_2_, 64%; 3. PhCHO, NBS, Et_2_N, MeOH, 33%.

In 2008, Nakahara et al. reported the synthesis of a carbocyclic spiroimidazoline from d-glucose [98]. Nucleophilic addition of dichloromethyllithium to ketone **226** afforded branched cyclopentitol **227**, which was then converted to azido aldehyde **228** on treatment with sodium azide (Scheme 44). Introduction of the second nitrogen atom of the imidazoline ring was achieved by reductive amination of **228**. After hydrogenation, condensation of the resulting diamine **229** with benzaldehyde using *N*-bromosuccinimide gave desired imidazoline **190**.

Other carbohydrate derivatives containing spirocycles in the anomeric position described in the literature include spirolactones [99], spiroaminals [100,101], spiroazacycles [102] and spirosulfamidates [103]. As their structural resemblance to spironucleosides is just anecdotal, these derivatives are outside of the scope of this review. 

## 6. Polycyclic Spironucleosides

**Figure 10:**
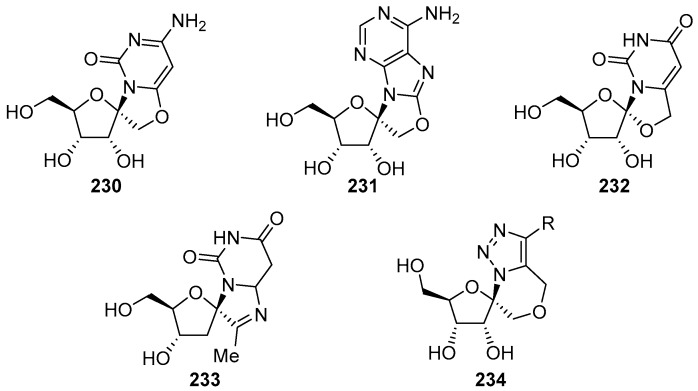
Polycyclic spironucleosides.

A number of spironucleosides in which the nucleobase is attached to the anomeric position of the sugar giving rise to a polycyclic system have been described. Such molecules provide conformationally fixed models, which can be useful to elucidate the glycosidic torsion angle of nucleosides (Figure 10).

**Scheme 45:**
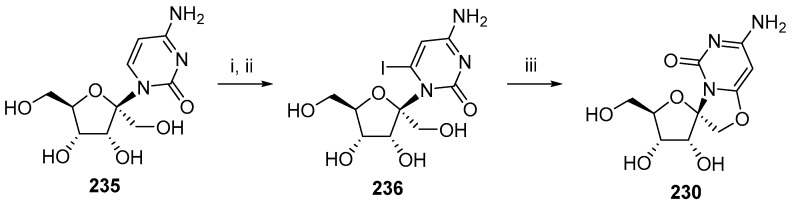
Reagents and conditions: (i) Hg(OAc)_2_, ∆; (ii) iodomercuration, 75%; (iii) *t*-BuOK, DMSO, 60 °C, 60%.

Early work from Zavgorodny resulted in the development of two synthetic routes to prepare *syn*-like anhydronucleosides modified at the C-1′ position of the 2-α-d-psicofuranosides [104]. Heating the starting compound psicofuranosyl cytosine **235** [105] with a solution of mercury (II) acetate followed by iodomercuration of the resulting intermediate afforded the iododinated compound **236**, which was then converted on nucleoside **230** on treatment with potassium *tert*-butoxide in DMSO (Scheme 45).

**Scheme 46:**
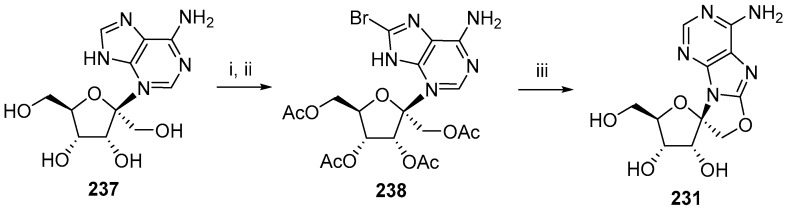
Reagents and conditions: (i) Ac_2_O, Py, 93%; (ii) Br_2_, EtOH, 63%; (iii) NH_3_, MeOH, 51%.

On the other hand, starting from psicofuranosyl nucleoside **237**, acetylation and bromination gave intermediate **238**, which on treatment with methanolic ammonia afforded the cyclonucleoside **239** (Scheme 46).

**Scheme 47:**
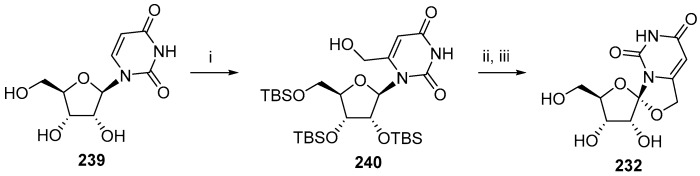
Reagents and conditions: (*i*) 1. Bu^t^Me_2_SiCl, imidazole, DMF, r.t., 16 h. 2. LDA, THF, −70 °C, 3 h, HCO_2_Et, −0 °C, 2 h. 3. NaBH_4_, MeOH, r.t., 30 min, 68%; (ii) PhI(OAc)_2_, I_2_, cyclohexane, *h*v, 28 °C, 5 h; (iii) Bu_4_NF on SiO_2_, THF, r.t., 2 h, 90%.

Gimisis and collaborators reported the synthesis of a spyronucleoside containing a remarkably stable orthoamide modification of the C-1′ anomeric position [106]. Starting from uridine **239** [107], silylation and hydroxymethylation afforded compound **240**, which under Suárez conditions afforded, after deprotection of the silyl groups, derivative **232** (Scheme 47).

**Scheme 48:**
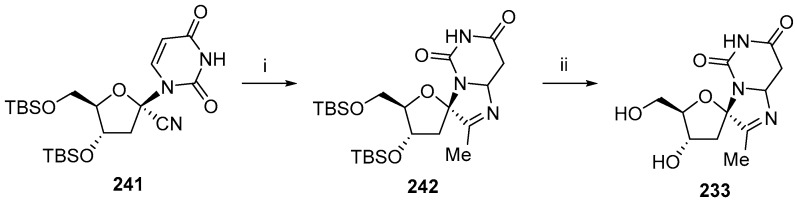
Reagents and conditions: (i) MeLi, THF, −8 °C, 20 min, 52%; (ii) NH_4_F, MeOH, reflux, 14 h, 90%.

The same researchers also described a synthesis of polycyclic spironucleosides based on the reaction of l′-*C*-cyano-pyrimidine nucleosides and organolithium reagents [108,109]. Thus, reaction of nucleoside **241** with methyllithium afforded, under the appropriate experimental conditions, spironucleoside **242** (Scheme 48). Then, deprotection of compound **242** by overnight treatment with ammonium fluoride in refluxing MeOH finally gave the corresponding desilylated nucleoside **233**.

**Scheme 49:**
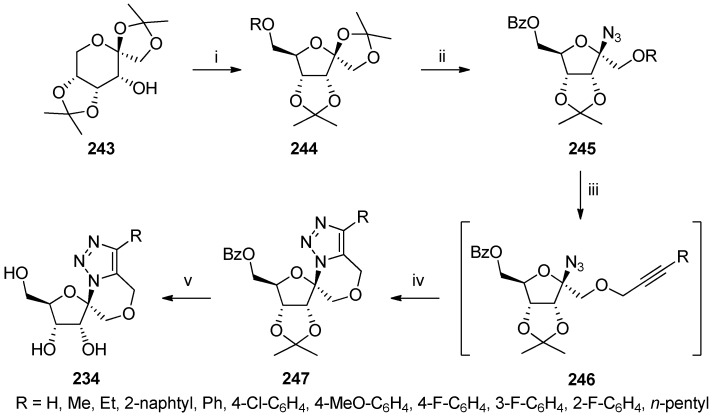
Reagents and conditions: (i) Amberlyst A15, acetone; (ii) 1. BzCl, Et_3_N, DMAP, DCM, 0 °C to r.t. 2. TMSN_3_, TMSOTf, 4Å MS, CH_3_CN, 0 °C, 5 min; (iii) 1. AcOH, MeOH, acetone; 2. propargyl bromide, CH_3_CN, 0 °C, 2 h; (iv) toluene, reflux, 24 h; (v) 1. NH_3_, MeOH; 2. Dowex-H^+^, MeOH/H_2_O 8:2, 50 °C.

Dell’Isola et al. recently reported the synthesis of bioactive spirocyclic triazolooxasine nucleosides [110]. The synthetic route started from the isomerization of the d-psicopyranose derivative **243** [111] into the furanose form **244**, promoted by an Amberlyst acid resin in acetone (Scheme 49). 

Benzoylation of **244**, followed by treatment with azidotrimethylsilane in the presence of trimethylsilyl triflate in acetonitrile to afforded compound **245**. After acidic hydrolysis of the silyl protecting group, *O*-alkylation of compound with a range of propargyl bromides afforded a series of propargyl ether intermediates **246**, which underwent intramolecular 1,3-dipolar cycloaddition achieved a novel library of protected anomeric spironucleosides **247**. Deacylation of **247**, followed by hydrolysis of the isopropylidene group, yielded finally anomeric spironucleosides **234**.

## 7. Conclusions

In summary, this literature review reports on all synthetic approaches to hydantocidin and their analogues and also to similar classes of spironucleosides such as diketopiperazines or barbiturates. Taking into account the biological relevance of spironucleosides, these derivatives were the synthetic target of many researchers since the pioneering work by Mio et al. Most of the reported syntheses of spironucleosides have revolved around the use of sugar derivatives as starting materials to generate the desired stereochemistry of the hydroxyl groups In this regard, the extensive work by Fleet and co-workers on the preparation of sugar amino acids (SAAs) and their transformation into spironucleosides provided tremendous advances for the future development of this fascinating class of biologically active compounds. However, much work remains to be done, as more spironucleoside analogues are still required for further structure-activities studies. Looking to the future, the widespread field of application of spironucleosides in medicinal chemistry, and the emergence of increasingly sophisticated synthetic methodologies, will certainly ensure continued interest in the development of this class of “synthetic” nucleosides.
